# Excited States, Symmetry Breaking, and Unphysical Solutions in State-Specific CASSCF Theory

**DOI:** 10.1021/acs.jpca.3c00603

**Published:** 2023-05-04

**Authors:** Antoine Marie, Hugh G. A. Burton

**Affiliations:** † Physical and Theoretical Chemical Laboratory, Department of Chemistry, University of Oxford, Oxford OX1 3QZ, U.K.

## Abstract

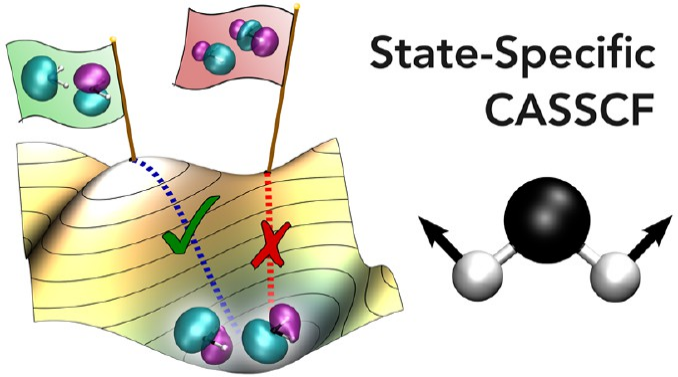

State-specific electronic structure theory provides a route toward balanced excited-state wave functions by exploiting higher-energy stationary points of the electronic energy. Multiconfigurational wave function approximations can describe both closed- and open-shell excited states and avoid the issues associated with state-averaged approaches. We investigate the existence of higher-energy solutions in complete active space self-consistent field (CASSCF) theory and characterize their topological properties. We demonstrate that state-specific approximations can provide accurate higher-energy excited states in H_2_ (6-31G) with more compact active spaces than would be required in a state-averaged formalism. We then elucidate the unphysical stationary points, demonstrating that they arise from redundant orbitals when the active space is too large or symmetry breaking when the active space is too small. Furthermore, we investigate the singlet–triplet crossing in CH_2_ (6-31G) and the avoided crossing in LiF (6-31G), revealing the severity of root flipping and demonstrating that state-specific solutions can behave quasi-diabatically or adiabatically. These results elucidate the complexity of the CASSCF energy landscape, highlighting the advantages and challenges of practical state-specific calculations.

## 1 Introduction

Electronic excited states are fundamentally higher-energy solutions to the time-independent Schrödinger equation. “State-specific” (SS) representations can be identified using higher-energy stationary points of the electronic energy landscape.^1^ The exact excited states in full configuration interaction (FCI) correspond to energy saddle points, and the number of downhill Hessian eigenvalues increases with each energy level.^1−7^ Higher-energy stationary points also exist in nonlinear wave function approximations, but the development of practical state-specific methods has been hindered by the challenges of non-ground-state optimization, the nonlinearity of the electronic energy landscape, and the presence of unphysical solutions.

Instead, the workhorse of modern excited-state electronic struture theory is linear-response time-dependent density functional theory (LR-TDDFT), which predicts excitation energies from the response of the ground-state electron density to a weak external perturbation.^8−10^ Despite its computational efficiency, LR-TDDFT inherits the failures of approximate Kohn–Sham (KS) exchange–correlation functionals, creating large errors for bond dissociation or open-shell electronic states.^11^ Furthermore, the ubiquitous adiabatic approximation excludes double excitations and their associated avoided crossings.^10,12^ Alternative single-reference methods, such as algebraic diagrammatic construction (ADC)^13,14^ and equation-of-motion coupled cluster (EOM-CC),^15,16^ can provide more accurate excitation energies at a greater computational cost but depend strongly on the quality of the reference determinant. The strong influence of the ground-state orbitals can also create an unbalanced description of charge transfer and Rydberg excitations,^17,18^ where significant electronic relaxation can occur.^8−10,19−21^

These challenges have encouraged researchers to revisit excited state-specific approximations. For higher-energy SCF calculations (ΔSCF), this progress has been catalyzed by the development of new optimization algorithms that avoid variational collapse to the ground state, including the maximum overlap method,^22−24^ square-gradient optimization,^25,26^ state-targeted energy projection,^27^ quasi-Newton direct orbital optimization,^28−30^ and generalized variational principles.^31^ Recent calculations have shown that higher-energy Hartree–Fock (HF) and KS-DFT solutions can accurately describe charge transfer and double excitations at a low computational cost.^22,26^ Beyond SCF approximations, higher-energy variational or projective coupled-cluster (ΔCC) solutions can provide more accurate double and double-core excitations by incorporating dynamic electron correlation.^32−38^ While ΔSCF and ΔCC are successful for double and charge transfer excitations, these single-reference methods cannot describe open-shell excited states and statically correlated ground states. The onset of this failure usually becomes apparent through spin contamination,^39,40^ spontaneous symmetry breaking,^11,23,39,41−45^ and additional unphysical solutions.^32−35,37−39^ Furthermore, the solutions of interest can disappear as the molecular structure changes, creating discontinuous excited-state energy surfaces or gradients.^38,39,46−50^

Multiconfigurational SCF (MCSCF) methods,^51^ particularly the complete active space self-consistent field (CASSCF) formulation,^52−54^ are the state of the art for describing statically correlated electronic systems.^55^ The CASSCF wave function is a linear expansion of all the configurations that can be constructed from a set of partially occupied “active orbitals”, and the energy is optimized with respect to the configuration interaction (CI) and orbital coefficients simultaneously.^53^ It has long been known that higher-energy MCSCF solutions can represent electronic excited states^56−61^ and that multiple symmetry-broken CASSCF solutions can occur for an inadequate active space.^62,63^ More recently, MCSCF expansions truncated to single excitations have shown promise for singly excited charge transfer states,^64−68^ while state-specific configuration interaction with higher degrees of truncation can handle challenging multireference problems and singly and doubly excited states.^69^ However, the strong coupling between the orbital and CI degrees of freedom makes the optimization challenging, and second-order optimization algorithms are generally required to reach convergence in practice.^70−83^

Extensive research in the 1980s focused on characterizing higher-energy MCSCF solutions. It was originally suggested that an *n*th excited state approximation should be the *n*th state in the configuration expansion.^73^ However, this requirement is often not achieved, resulting in “root flipping”.^2,56,75^ Furthermore, several stationary points satisfying this condition can often be identified.^3,5,84^ The enormous complexity of the multiconfigurational solution space led Golab et al. to conclude that “selecting an MCSCF stationary point is a very severe problem”.^3^ Instead, the state-averaged (SA) approach is generally used, where a weighted average energy of the *n* lowest CI states constructed from one set of orbitals is optimized.^75^ While this approach has become the method of choice for excited-state CASSCF, it has several disadvantages: discontinuities can occur on the SA-CASSCF potential energy surface if two states require orbitals with significantly different character;^85^ the number of states is limited by the size of the active space; large active spaces are required to target high-lying states; and the Hellmann–Feynman theorem cannot be applied to compute nuclear gradients because individual SA-CASSCF solutions are not stationary points of the energy.

Multiconfigurational linear response formalisms^86−88^ can also be applied to CASSCF reference wave functions to obtain excitation energies.^21,89^ This approach avoids the challenges of root flipping and can incorporate some state-specific orbital relaxation, generally resulting in more accurate energies than state-averaged formalisms.^21^ Furthermore, LR-CASSCF is capable of describing excitations that are “outside” the active space. However, as a linear response formalism, this approach is still limited to one-electron excitations relative to the ground state and will struggle for problems with a quasi-degenerate ground-state wave function.

Instead, the limitations of current excited-state CASSCF formalisms and the development of non-ground-state SCF optimization algorithms have inspired several new investigations into state-specific CASSCF excited states. In particular, Neuscamman and co-workers have developed generalized variational principles^90,91^ and the *W*Γ approach inspired by MOM-SCF,^92^ demonstrating that the issues of root flipping and variational collapse to the ground state can be successfully avoided. Despite these advances, we still do not have a complete understanding of the multiple stationary points on the SS-CASSCF energy landscape, and several practical questions remain. For example, how many stationary points are there, and how does this change with the active space or basis set size? Where do unphysical solutions arise, what are their characteristics, and when does symmetry breaking occur? And finally, do state-specific excitations behave diabatically or adiabatically as the molecular structure evolves?

In this work, our aim is to answer these questions and establish a theoretical foundation for practical excited state-specific calculations. Using second-order optimization techniques, we investigate the existence and properties of multiple CASSCF solutions in typical molecular systems. Our numerical optimization exploits analytic gradients and second derivatives of the CASSCF energy, and the relevant differential geometry is summarized below. Using these techniques, we comprehensively enumerate the multiple CASSCF solutions in H_2_ (6-31G) and characterize the resulting unphysical solutions. We find that state-specific calculations can accurately describe high-lying excitations with fewer active orbitals than state-averaged formalisms and reveal that multiple solutions can arise from active spaces that are too large or too small. We then investigate the singlet–triplet crossing in CH_2_ (6-31G) and the avoided crossing of LiF (6-31G), demonstrating the importance and difficulty of selecting the correct physical solution.

## 2 Methods

### 2.1 Defining the CASSCF Wave Function

A multiconfigurational wave function is defined as a linear combination of *M* many-body configurations:
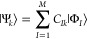
1where |Φ_*I*_⟩ are different configurations built from a common set of molecular orbitals (MOs) ϕ_*p*_(***x***) and the *C*_*Ik*_ are the variable CI coefficients for state *k*.^93^ Here, ***x*** = (***r***, σ) is the combined spatial and spin electronic coordinate. The configurations |Φ_*I*_⟩ may be defined as Slater determinants, which enable very efficient computational implementations,^94,95^ or configuration state functions (CSFs) that explicitly preserve the ⟨*Ŝ*^2^⟩ symmetry.^96^ Here we only consider the determinant-based expansion that is more common in current CASSCF implementations. The MOs are constructed as linear combinations of *n* (nonorthogonal) atomic orbitals (AOs) χ_μ_(***x***) as
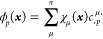
2where we use the nonorthogonal tensor notation of ref (97) and the *c*_·*p*_^μ·^ denote the variable MO coefficients. Normalization of the wave function and orthogonalization of the MOs are guaranteed by the constraints
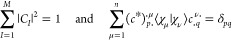
3where ⟨χ_μ_|χ_ν_⟩ are the AO overlap matrix elements. We will only consider wave functions where *C*_*Ik*_ and *c*_·*p*_^μ·^ are real.

When every electronic configuration for a finite basis set is included in an FCI expansion, the global minimum on the parametrized electronic energy landscape corresponds to the exact ground state.^1^ Excited states form saddle points of the energy, and the number of downhill directions increases with each excitation.^1,3,6,7^ The FCI wave function is invariant to unitary transformations of the MOs, but the number of configurations scales exponentially with the system size.

The complete active space (CAS) framework builds a truncated expansion using every configuration within a set of “active orbitals” that describe the dominant static electron correlation.^53^ The orbitals are partitioned into inactive and virtual orbitals that are doubly occupied or empty in every configuration, respectively, and active orbitals with varying occupations. Simultaneously optimizing the energy with respect to the orbital and CI coefficients leads to the state-specific CASSCF approach and gives true stationary points of the electronic energy.^53,54,74^ If the CASSCF wave function targeting the *k*th excited state is represented by the *k*th eigenstate of the corresponding CAS-CI expansion, then the Hylleraas–Undheim–MacDonald theorem^98,99^ also provides an upper bound to the excited-state energy.^3^

### 2.2 Differential Geometry of the CASSCF Energy

We exploit an exponential form of the CASSCF wave function that conserves the orthogonality constraints (eq 3).^70,72^ Starting from an initial CASSCF wave function |Ψ_0_⟩, an arbitrary step can be defined using unitary transformations as
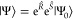
4where e^*R̂*^ and e^*Ŝ*^ account for orbital relaxation and transformations of the CI component, respectively. The *R̂* operator is anti-Hermitian and is defined using the second-quantized creation and annihilation operators for the current MOs as^70,100^
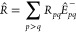
5where the spin-adapted one-body anti-Hermitian replacement operators are^93^
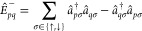
6The invariance of the energy with respect to inactive–inactive, active–active, and virtual–virtual orbital transformations means that *R*_*pq*_ can be further restricted to only excitations between different sub-blocks. Similarly, e*^Ŝ^* performs a unitary transformation between the CI component of |Ψ_0_⟩ and the remaining orthogonal states |Ψ_*K*_⟩ in the current CASCI space, with *Ŝ* defined as^72^
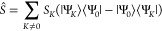
7Using the exponential parametrization, the CASSCF energy can be expressed as
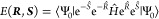
8where ***R*** and ***S*** are vectors that gather the *R*_*pq*_ and *S*_*K*_ coefficients in the orbital and CI transformations, respectively, and *Ĥ* is the electronic Hamiltonian. Stationary points of *E*, corresponding to optimal CASSCF solutions, then occur when the gradients with respect to orbital and CI transformations are simultaneously zero. Performing a Baker–Campbell–Hausdorff expansion of the energy to second order gives^72^
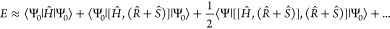
9Expressions for the first- and second-derivatives of the energy can then be identified as
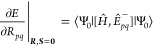
10a
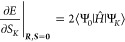
10band
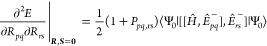
11a
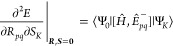
11b
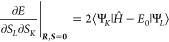
11cwhere *E*_0_ is the energy at ***R***, ***S*** = **0**, *P*_*pq*,*rs*_ permutes the (*pq*) and (*rs*) indices, and the Hermiticity of *Ĥ* and [*Ĥ*, *Ê*_*pq*_^–^] have been exploited. Explicit formulas for these expressions have been summarized elsewhere (see ref (4)) but are given in Supporting Information (SI) section S1 for completeness.

Note that the first and second derivatives can only be computed when ***R*** = **0** and ***S*** = **0**.^100^ Therefore, after taking a step in the parameter space, the energy gradient and Hessian must be computed using the new MOs and CI vectors corresponding to the updated wave function. A similar shift in the reference state after each step is also required for second-order HF optimization algorithms.^39,101^

### 2.3 Characterizing Distinct Solutions

The invariance to unitary transformations within each orbital partition means that the same CASSCF wave function can be identified with different CI or MO coefficients. We use the overlap between two stationary solutions |^*x*^Ψ⟩ and |^*w*^Ψ⟩ to define a positive-semidefinite distance metric:
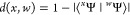
12The overlap for two arbitrary CI wave functions with *M*_*x*_ and *M*_*w*_ configurations, respectively, is given by
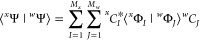
13Since |^*x*^Ψ⟩ and |^*w*^Ψ⟩ have different sets of MOs, evaluating the overlap matrix elements ⟨^*x*^Φ_*I*_|^*w*^Φ_*J*_⟩ requires a nonorthogonal framework. We compute these matrix elements using the extended nonorthogonal Wick’s theory,^102,103^ which avoids the computationally expensive generalized Slater–Condon rules.^104^

To understand the MOs in a CASSCF solution, we canonicalize the inactive and virtual orbitals and construct natural orbitals within the active space. The canonical inactive and virtual orbitals and their associated orbital energies are identified by diagonalizing the relevant sub-blocks of the Fock matrix, defined as^93^
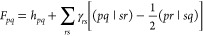
14where γ_*pq*_ are the one-body reduced density matrix elements in the MO basis, *h*_*rq*_ are the one-electron Hamiltonian matrix elements, and (*pq*|*rs*) are the two-electron repulsion integrals. The natural orbitals within the active space are the eigenvectors of the one-body reduced density matrix, and their eigenvalues are the occupation numbers *n*_*p*_.^105^

### 2.4 Optimization Techniques

Since we are concerned with understanding the CASSCF solution space, we require an algorithm capable of converging arbitrary stationary points on the energy landscape, including minima and higher-index saddle points. Higher-energy CASSCF stationary points are notoriously difficult to converge due to the strong coupling between the orbital and CI degrees of freedom^56,60,61,72,77^ and the possibility of root flipping in the configuration space.^75,106^ Therefore, we employ second-order techniques that introduce the orbital–CI coupling through the analytic Hessian matrix of second derivatives. Very recent algorithmic developments have shown that genuine second-order optimization can be applied to large molecular systems and can converge challenging cases where gradient-based first-order optimization fails.^107,108^ For our purposes, full second-order optimization allows us to systematically explore and characterize the state-specific CASSCF energy landscape with confidence.

We search for multiple solutions using several initial guesses generated using random orbital and CI rotations from the ground-state HF solution. The eigenvector-following technique with analytic gradient and Hessian information was used to target stationary points with a particular Hessian index.^109,110^ While this method has been described in detail elsewhere (see ref (111)), we include a summary in SI section S2. Related mode-following methods have previously been applied to locate higher-energy electronic stationary points in multiconfigurational^2−4,112^ and single-determinant^39^ SCF calculations. The convergence behavior was further improved with a modified trust region approach based on the dogleg method.^113^ Trust region methods are a well-established approach for controlling the convergence of second-order methods in CASSCF calculations.^77−80,107,108,114^ Once a set of stationary points have been identified, their evolution with changes in the molecular structure can be determined by using the optimized orbital and CI coefficients at one geometry to define an initial guess at the next geometry. Since the Hessian index may not be conserved along a reaction coordinate,^2^ these subsequent calculations are performed using a trust region Newton–Raphson algorithm, as described in SI section S3.

We have implemented this numerical optimization in an extension to the PySCF software package.^115^ Our approach employs a determinant-based expansion of the CAS-CI wave function without any constraint on the total spin ⟨*Ŝ*^2^⟩. As we shall demonstrate in section 3.2, this approach can result in spin-contaminated wave functions that are genuine stationary points of our CASSCF parametrization. The convergence threshold for the root-mean-square value of the gradient amplitudes was universally set to 10^–8^ E_h_. The canonical and natural orbitals for stationary points were subsequently computed using PySCF and visualized using VMD.^116^ All other graphical figures were created using Mathematica 12.0.^117^

## 3 Results and Discussion

### 3.1 Molecular H_2_ Dissocation

**Figure 1:**
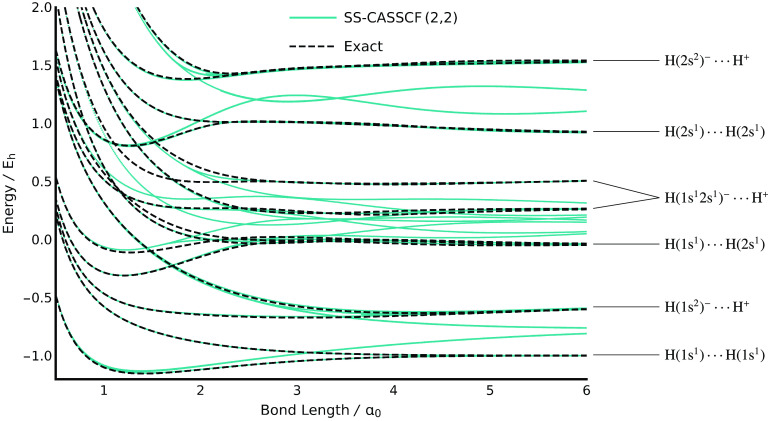
State-specific CASSCF(2,2) stationary points can be identified for every excited FCI state in H_2_. Additional solutions can also be found that dissociate to an unphysical electronic state.

We start by considering the H_2_ binding curve using the 6-31G basis set.^118^ To identify all the CASSCF(2,2) solutions, a comprehensive search was performed using up to 1000 random starting points for target Hessian indices from 0 to 16. Solutions were identified near the equilibrium geometry *R* = 1.0 *a*_0_ and the dissociation limit *R* = 6.0 *a*_0_ and were then traced over all bond lengths, as shown in Figure 1. We believe that we have found every stationary point on the landscape, although the nature of nonconvex optimization means that this can never be guaranteed. To the best of our knowledge, this study is the first comprehensive enumeration of the CASSCF solutions for a molecular system.

#### 3.1.1 Excitations Near Equilibrium

Near the equilibrium geometry, the ground state of H_2_ can be accurately described using a single-reference approximation. We have identified 25 stationary points on the CASSCF(2,2) energy landscape using the 6-31G basis set, corresponding to 19 singlet solutions and six triplet solutions (Table I). Of course, this number will increase for larger basis sets, as there will be more excited states, although we show in section 3.1.2 that a larger basis set can also give more unphysical solutions (Table II). Each of the exact FCI states has a corresponding SS-CASSCF(2,2) counterpart, and the energetic agreement between these solutions is consistent for all excitations. We have also found several additional solutions that appear to be less accurate approximations to the exact states, which will be characterized in sections 3.1.2 and 3.1.3. In comparison, the SA-CASSCF(2,2) approach can only describe the lowest triplet and the three lowest singlet states, while increasing the number of active orbitals to a (3,2) active space provides an approximation to the lowest nine excitations.

**Table I:** Energies of H_2_ at *R* = 1 *a*_0_ Using the 6-31G Basis Set for Various Formalisms: FCI, SA-CASSCF(2,2), SA-CASSCF(3,2), and SS-CASSCF(2,2)

State	FCI	SA(2,2)	SA(3,2)	SS(2,2)	⟨*S*^2^⟩	Index
0	–1.09897	–1.07170	–1.08924	–1.09225	0	0
				–1.08569	0	1
				–1.07871	0	2
1	–0.57616	–0.57166	–0.57406	–0.57417	2	1
2	–0.46395	–0.43494	–0.44196	–0.46368	0	2
3	–0.28180		–0.27990	–0.27990	2	2
4	–0.07450		–0.06164	–0.05946	0	3
5	0.32015	0.33066	0.32624	0.31914	0	3
				0.31821	0	2
				0.31844	0	2
				0.32440	0	3
6	0.51519		0.51654	0.51638	2	3
7	0.57224		0.61682	0.61429	0	4
8	0.62520			0.62401	2	3
9	0.86353		0.86876	0.86392	0	5
				0.85673	0	4
				0.86266	0	4
10	0.96373			0.91147	0	4
11	1.30761			1.30572	2	4
12	1.46479			1.45704	0	5
13	1.61884			1.61685	2	5
14	1.81277			1.80747	0	6
15	2.71948			2.71766	0	7
				2.70046	0	6
				2.69883	0	5

These results demonstrate two important features of state-specific calculations. First, they can describe more excited states than state-averaged calculations by defining the active space using only orbitals that are relevant for a particular excitation. This property allows higher-energy excitations to be predicted while avoiding large active spaces and the associated increase in the configuration space. An upper bound to the exact excited-state energy is only provided by stationary points that correspond to the correct excitation within the CASCI configuration space,^3^ although more accurate energies are generally preferred even if they are not variational. Second, bespoke orbital optimization for each state-specific solution can give more accurate total energies for the excited states compared to the state-averaged approach. For example, the mean absolute deviations (MADs) for the lowest four states are 2.5 and 17.8 mE_h_ for the state-specific and state-averaged CASSCF(2,2) approaches, respectively.

Using analytic second derivatives of the energy also allows the nature of SS-CASSCF(2,2) stationary points to be characterized according to their number of downhill directions. The corresponding Hessian index for each solution is listed in Table I. It is known that the exact *n*th excited state should have *n* downhill directions.^1,2,4^ We find that the SS-CASSCF(2,2) excited states are all saddle points on the electronic energy landscape and that the Hessian index generally increases with the energy, in common with the observations for other theoretical approximations.^22,25,37,39^ However, except for the lowest three exact states, the Hessian index does not provide a reliable indicator of the corresponding exact excitation index. This mismatch must always occur for higher-lying excited states, as the approximate CASSCF(2,2) wave function has fewer degrees of freedom than the exact formulation. Consequently, if we only consider stationary points of the correct Hessian index, then we must forego the advantages of capturing state-specific excitations outside the state-averaged active space.

#### 3.1.2 Multiple Ground-State Solutions

While Table I shows that a SS-CASSCF(2,2) approximation can be identified for each exact eigenstate, we also find additional state-specific solutions. In particular, there are three close-lying stationary points that can be considered as approximations to the ground state, with Hessian indices of 0, 1, and 2 in order of ascending energy. This pattern of multiple solutions is repeated for the (2σ_g_)^2^ and (2σ_u_)^2^ singlet configurations, while the other closed-shell (1σ_u_)^2^ configuration exhibits four close-lying solutions. Choosing the most physical solution for each eigenstate presents a challenge for state-specific CASSCF approaches. Therefore, it is important that we understand their mathematical origins and physical differences.

**Figure 2:**
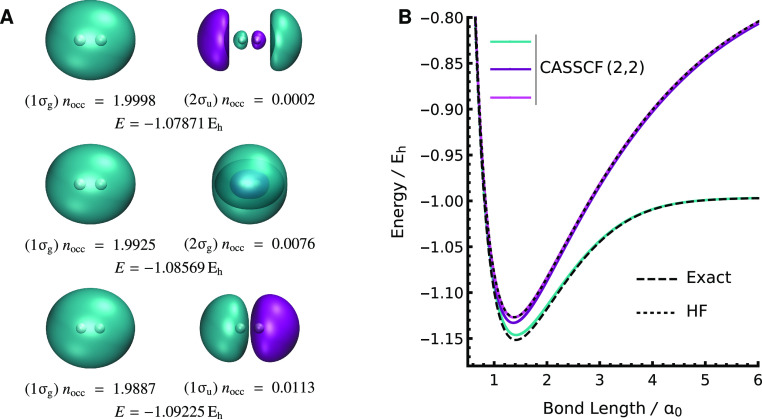
There are three SS-CASSCF(2,2) solutions that represent the exact ground state in H_2_. (A) Comparison of the natural orbitals for each ground-state solution at *R* = 1.0 *a*_0_. (B) Only the lowest-energy solution dissociates correctly, while the higher-energy solutions mirror the restricted Hartree–Fock binding curve.

The natural orbitals in the active space provide a clear explanation for the multiple H_2_ ground-state solutions. Figure 2A compares the natural orbitals and occupation numbers for the three lowest-energy singlet stationary points. Since the ground state at the equilibrium geometry can be relatively well approximated by a single closed-shell Slater determinant, the active space for each of these solutions includes a (1σ_g_)-like natural orbital that is almost completely doubly occupied. This natural orbital dominates the electronic wave function, and the corresponding energies are all relatively close approximations to the exact ground state. However, the second active orbital, which is almost completely unoccupied, is different for each solution, corresponding to a (1σ_u_), (2σ_g_), or (2σ_u_) orbital as the energy increases, respectively. These higher-energy stationary points have downhill orbital rotations that interconvert the multiple ground-state solutions and correspond to the negative eigenvalues of the Hessian.

Different choices for the nearly unoccupied active orbital have only a small effect on the total H_2_ energy near equilibrium. However, the incorrect choice of the active space becomes very significant as the bond is stretched toward dissociation. Only the {1σ_g_, 1σ_u_} active space can correctly dissociate into the H(1s)···H(1s) ground state of the dissociated fragments (Figure 2B). In contrast, the binding curves for the {1σ_g_, 2σ_g_} and {1σ_g_, 2σ_u_} solutions mirror the RHF energy, as the corresponding wave functions are close to a single Slater determinant at all geometries, with (1σ_g_) occupation numbers at dissociation of 1.997 and 1.999, respectively. Notably, the stationary points preserve the character of the active orbitals along the potential energy surface, suggesting that SS-CASSCF solutions exhibit some degree of diabatic character.

The same pattern of solutions is observed for the other closed-shell solutions. However, the (1σ_u_)^2^ configuration exhibits an additional multiple solution where the nearly unoccupied active orbital corresponds to a symmetry-broken 2s-like orbital localized on either the left or right H atom. This symmetry breaking results in a twofold-degenerate pair of stationary points.

These results indicate that additional solutions can arise from the free choice of virtual orbitals when the active space is larger than required for the degree of static correlation. Malrieu and coworkers elegantly summarized this phenomenon by stating that “the so-called valence CASSCF wave function does not necessarily keep a valence character when the wave function concentrates on a closed-shell valence bond structure”.^119^ Therefore, we expect that the number of ground-state solutions will increase combinatorially with the number of active orbitals or the basis set size, and the number of unphysical solutions can grow for larger active spaces even though the correct ground-state solution will become more accurate. Table II demonstrates this increase for H_2_ using the 6-311G basis set with three basis functions for each hydrogen atom,^120^ which gives five ground-state solutions for the (2,2) active space compared to the three found using 6-31G. Furthermore, in the (3,2) active space, there are two redundant active orbitals beyond the 1σ_g_ orbital that must be chosen from the five remaining orbitals, giving a total of 10 solutions through the binomial coefficient . The relative energy ordering of these additional solutions will depend on the amount of dynamic correlation captured by the redundant active orbitals, which may not correspond with the same orbital required to capture the static correlation in the dissociation limit. This phenomenon has previously been described for MgO, where oxygen-centered orbitals are preferred over the magnesium d orbitals,^91^ and transition metal compounds, where nonvalence d orbitals may be preferred over certain valence d orbitals.^121^

**Table II:** Close-Lying Ground-State (*n*,2) SS-CASSCF Energies (in E_h_) of H_2_ at *R* = 1 *a*_0_ Using the 6-311G Basis Set for Various Active Space Sizes *n*

SS(1,2): HF	–1.08025				
					
SS(2,2)	–1.09429	–1.08866	–1.08074	–1.08033	–1.08026
					
SS(3,2)	–1.10195	–1.09500	–1.09436	–1.09429	–1.08904
	–1.08886	–1.08867	–1.08082	–1.08075	–1.08034
					
SS(4,2)	–1.10251	–1.10212	–1.10196	–1.09507	–1.09500
	–1.09437	–1.08923	–1.08905	–1.08886	–1.08083
					
SS(5,2)	–1.10267	–1.10251	–1.10213	–1.09507	–1.08924
					
SS(6,2): FCI	–1.10267				

#### 3.1.3 Open-Shell Singlet and Triplet Excitations

**Figure 3:**
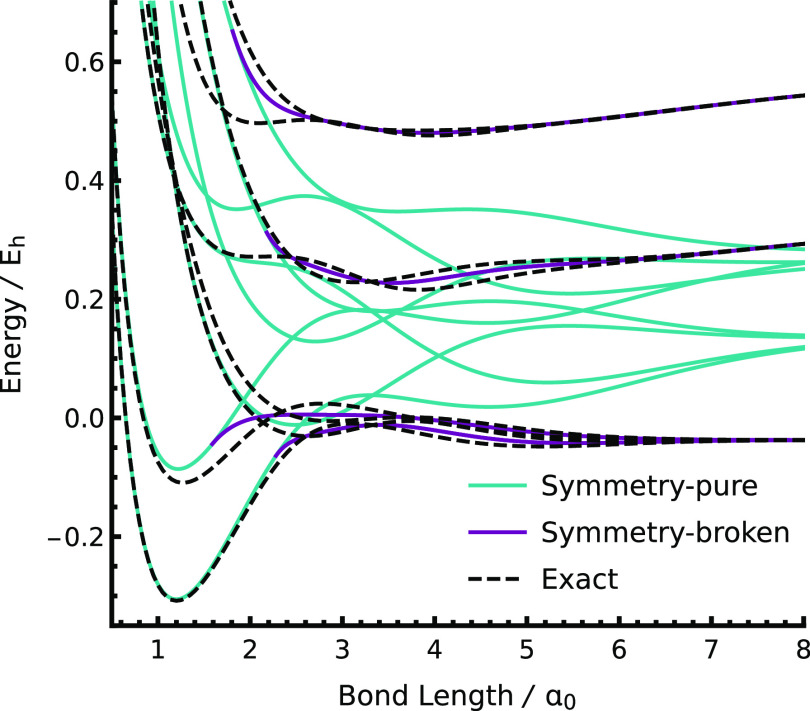
Spontaneous symmetry breaking occurs when the active space is not large enough to capture all the important configurations in the physical wave function, as illustrated for the 1s2s states in the dissociation of H_2_ (6-31G).

The low-lying open-shell triplet and singlet (1σ_g_)^1^(1σ_u_)^1^ configurations are represented by only one SS-CASSCF(2,2) solution across the full binding curve (Figure 1). These single solutions arise because all the active orbitals are required to describe the two-configurational static correlation and there is no flexibility for multiple solutions to exist. In addition, SS-CASSCF(2,2) gives an accurate representation of the open-shell (1σ_g/u_)^1^(2σ_g/u_)^1^ configurations. However, the accuracy of these solutions deteriorates in the dissociation limit, where additional symmetry-broken solutions can be identified (Figure 3). These additional solutions break spatial symmetry and spontaneously appear at instability thresholds that are multiconfigurational analogues to the Coulson–Fischer points^41^ in HF theory.^42,122−125^ Each stationary point is a pure singlet or triplet state and has a twofold degeneracy, reflecting the left–right symmetry of the molecule.

The origin of this symmetry breaking is explained by considering the correlation processes involved in the excited dissociation limit. These excited states dissociate to hydrogenic (1s)^1^(2s)^1^ configurations, where the occupied 1s and 2s orbitals can either be on the same or different atomic centers. Taking the latter case as an example, the corresponding open-shell singlet wave function at large nuclear separations has the form
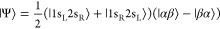
15Correctly describing this wave function requires an active space with four spatial orbitals {1s_L_, 1s_R_, 2s_L_, 2s_R_}, or equivalently {1σ_g_, 1σ_u_, 2σ_g_, 2σ_u_}, and thus, the SS-CASSCF(2,2) approximation is insufficient for these correlation mechanisms. Instead, the symmetry breaking reduces the SS-CASSCF(2,2) wave function to a subset of the dominant configurations, e.g.,
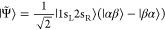
16The CASSCF configurations corresponding to each symmetry-broken solution are assigned in Table III. This “pinning” of the wave function onto a particular electronic configuration is directly analogous to the symmetry breaking phenomena observed in HF theory^126,127^ and demonstrates that the active space is too small to fully account for the static correlation.

**Table III:** Symmetry-Broken CASSCF(2,2) Solutions in the Dissociation of H_2_ are Twofold-Degenerate and Represent Dominant Configurations in the Exact Excitations

State	Energy/E_h_	⟨*S*^2^⟩	Configuration
A	0.543355	0.00	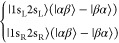
B	0.293363	2.00	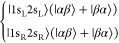
C	–0.037221	0.00	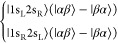
D	–0.037499	2.00	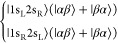

From the energy landscape perspective, the onset of symmetry-broken CASSCF(2,2) states is associated with a change in the Hessian index for the associated symmetry-pure solutions. For example, the symmetry-broken state D (Table III) emerges from the symmetry-pure (1σ_g_)^1^(2σ_g_)^1^ triplet state at an instability threshold close to *R* = 2.28 *a*_0_. The Hessian index of the symmetry-pure state changes from 2 to 3 at this point, while the symmetry-broken solutions form index-2 saddle points, leading to a higher-index analogue of a cusp catastrophe.^39,40,128^ Practically, the emergence of a zero Hessian eigenvalue at these instability thresholds may hinder the numerical optimization of second-order techniques onto these higher-energy stationary points. It is also interesting to note that while the symmetry-broken solutions describe two degenerate FCI states at dissociation, they only connect to one of the corresponding symmetry-pure solutions in the equilibrium region. Consequently, one cannot rely on these additional solutions to obtain an accurate and continuous representation of every excited state across all geometries.

### 3.2 Singlet–Triplet Crossing in Methylene

We next consider the bending mode of methylene, which has a diradical ground state with ^3^B_1_ symmetry and a low-lying 1^1^A_1_ excited state. The bond length was fixed to the value *R*(C–H) = 2.11 *a*_0_ identified by Bauschlicher and Taylor,^129,130^ and the 6-31G basis set was used.^118^ Methylene has a long history as a benchmark for electronic structure theory.^131^ One of the primary questions is the description of the singlet–triplet crossing between the low-lying ^3^B_1_ and 1^1^A_1_ states.

#### 3.2.1 Local Minima for the Minimal (2,2) Active Space

**Figure 4:**
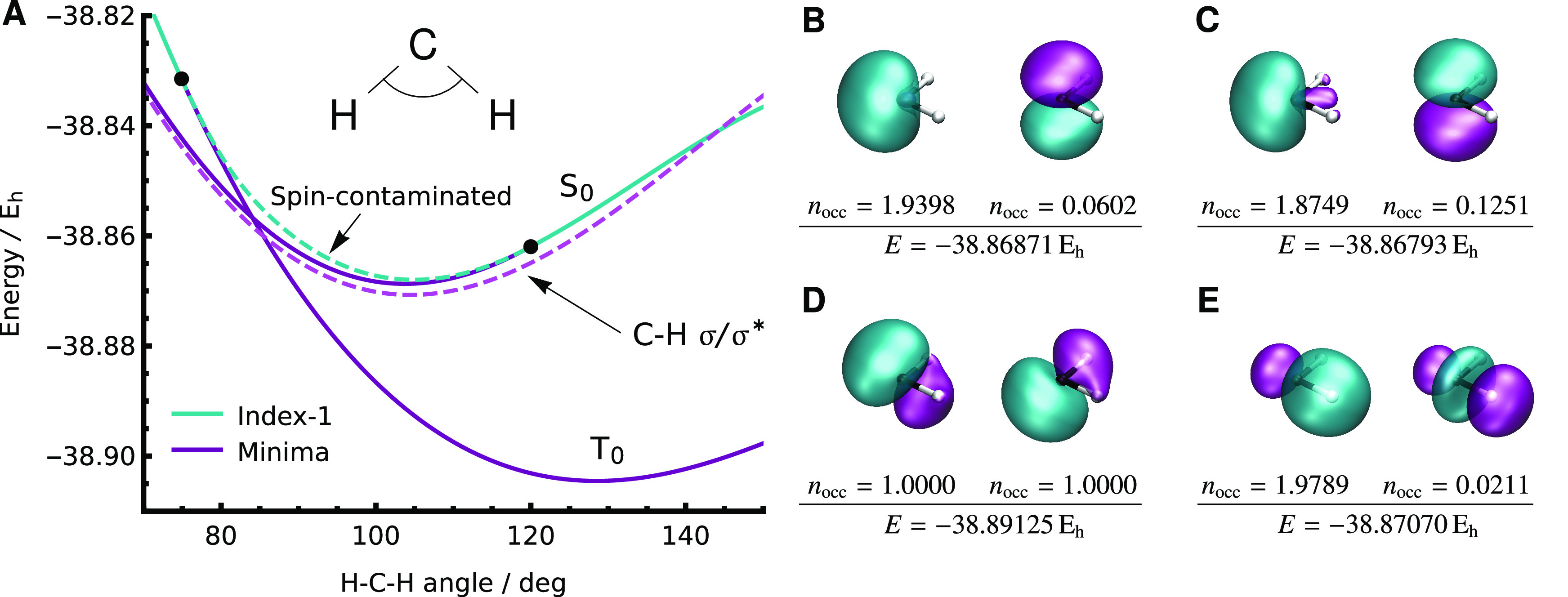
Low-lying SS-CASSCF(2,2) states in the bending mode of methylene representing the 1^3^B_1_ and 1^1^A_1_ configurations. (A) Both states remain local minima (solid purple) for a short region beyond the singlet–triplet crossing before becoming an index-1 saddle point (solid cyan). An additional spin-contaminated index-1 saddle point (dashed cyan) connects the two instability thresholds (black dots). Two degenerate local minima exist everywhere along the bending curve (dashed purple) with an active space containing C–H bonding σ and antibonding σ* orbitals. (B–E) The natural orbitals at a bond angle of 103.7° are illustrated for each solution.

A minimal two-configuration wave function is required to qualitatively describe both the lowest-energy singlet S_0_ (^1^A_1_) and diradical triplet T_0_ (^3^B_1_) states.^129^ Therefore, we begin by analyzing the SS-CASSCF(2,2) energy landscape. The S_0_ and T_0_ states are the ground states for small and large bond angles, respectively, and provide an example of a singlet–triplet crossing separating the two regimes. At bond angles of 76°, 102°, and 130°, a large number of stationary points can be identified with a variety of Hessian indices. Therefore, we simplify our analysis by focusing on a subset of low-energy solutions that resemble the desired physical states (Figure 4).

The energetic minimum of the S_0_ state occurs at a bond angle of 103.7°. While the S_0_ state is the first excited state at this geometry, we find that the corresponding SS-CASSCF(2,2) stationary point is a local minimum rather than an index-1 saddle point. This incorrect Hessian index arises from a root flip in the configuration space, where the singlet state is the ground state for the corresponding active orbitals. When the bond angle increases, this singlet state eventually becomes an index-1 saddle point. Similarly, when the bond angle decreases from 103.7°, the T_0_ state remains a local minimum beyond the point where it becomes the first excited state. This process behaves like an unphysical hysteresis, where the ground state remains a local minimum for a small region after a crossing point before becoming an index-1 saddle point at an instability threshold.

An additional index-1 saddle point can be identified that connects these two solutions and coalesces with each local minimum at the two instability thresholds. This unphysical index-1 stationary point is twofold-degenerate, has symmetry-pure spatial orbitals, but is spin-contaminated with an ⟨*Ŝ*^2^⟩ value that changes continuously from 0 to 2 as it connects the singlet and triplet states. Similar patterns of coalescing solutions have been observed in single-determinant SCF approximations,^40,122,132,133^ particularly in the generalized HF representation of a crossing between states with different ⟨*Ŝ*_*z*_⟩ values.^134^

In contrast to symmetry-broken SCF orbitals, the spin contamination observed here arises from mixing singlet and triplet states in the configurational part of the wave function. Therefore, although the current implementation employs a determinant-based expansion, this spin contamination will still occur using a linear combination of all configuration state functions with ⟨*Ŝ*_*z*_⟩ = 0 rather than a determinant-based expansion. Spin contamination could be avoided explicitly by including a spin-penalty function^135^ or if the CASSCF wave function is constructed from a linear combination of CSFs with the desired ⟨*Ŝ*^2^⟩ value.^136−142^

Since the S_0_ solution has only one significantly occupied active orbital, we predict the existence of closely related solutions that have alternative redundant orbitals with *n*_occ_ ≈ 0. Indeed, there are a pair of degenerate local minima that lie slightly lower in energy than the S_0_ solution. In contrast to the H_2_ ground state, including the inactive space means that methylene has multiple doubly occupied orbitals, and thus, the active orbital with *n*_occ_ ≈ 2 may also change between different solutions. The active orbitals for these symmetry-broken solutions are localized bonding σ and antibonding σ* orbitals for one of the two C–H bonds, and the degeneracy accounts for the two possible ways to localize onto one bond. Notably, the symmetry breaking here is associated with an active space that is too large, in contrast to H_2_, where symmetry breaking arises from an insufficient active space for the static correlation. These solutions are local minima across all the bond angles considered. While they provide an accurate energy for the S_0_ state near the singlet equilibrium structure, this deteriorates for large angles, as the active space cannot describe the diradical open-shell ^1^Σ_g_^+^ state at the linear geometry. Their existence indicates that the C–H σ/σ* configurations provide an important contribution to static correlation and should ideally be included in the active space, as suggested by Bauschlicher and Taylor.^129,130^

#### 3.2.2 Full Valence Active Space

**Figure 5:**
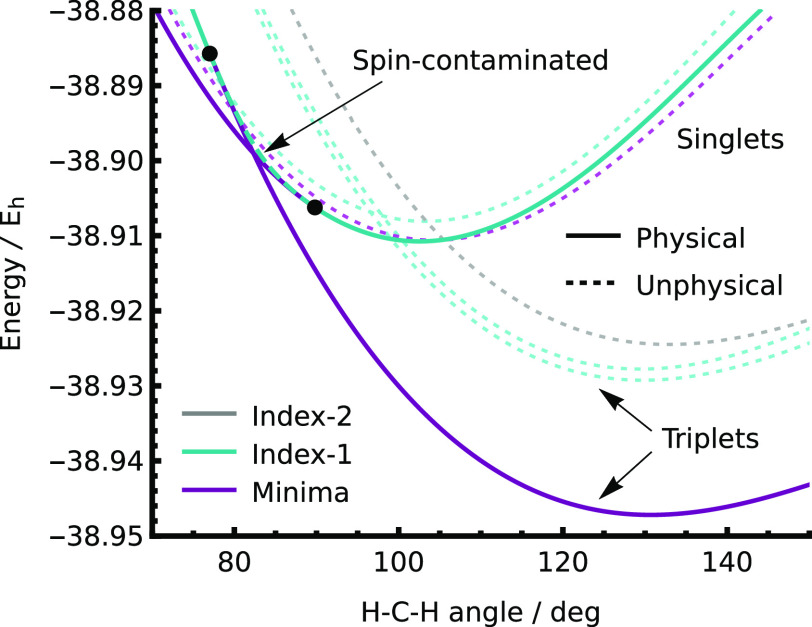
Low-lying SS-CASSCF(6,6) states for the bending mode of methylene representing the 1^3^B_1_ and 1^1^A_1_ configurations. The full-valence (6,6) active space introduces more unphysical solutions but does not remove the spin-contaminated solution that arises at the crossing point.

Using the full-valence (6,6) active space, we find that the symmetry-pure singlet state is now correctly represented by an index-1 saddle point at a bond angle of 102° (Figure 5). The unique downhill direction corresponds to a rotation in the configuration space only, as expected for the first excited state. Despite the larger active space, a root flip still occurs as the states approach the singlet–triplet crossing at 82.2°, with the singlet state becoming a local minimum at 89.6° and the triplet state becoming an index-1 saddle point at 77.0°. Like the (2,2) active space, a degenerate pair of unphysical, spin-contaminated index-1 saddle points connect the solutions that cross at the crossing point. This phenomenon occurs because the orbital optimization can lower the energy of the target excited state below that of the corresponding ground-state configuration when the energy gap becomes small. Therefore, while larger active spaces will reduce the range of molecular geometries affected, these unphysical local minima will be common for state-specific crossing points between different spin states.

**Figure 6:**
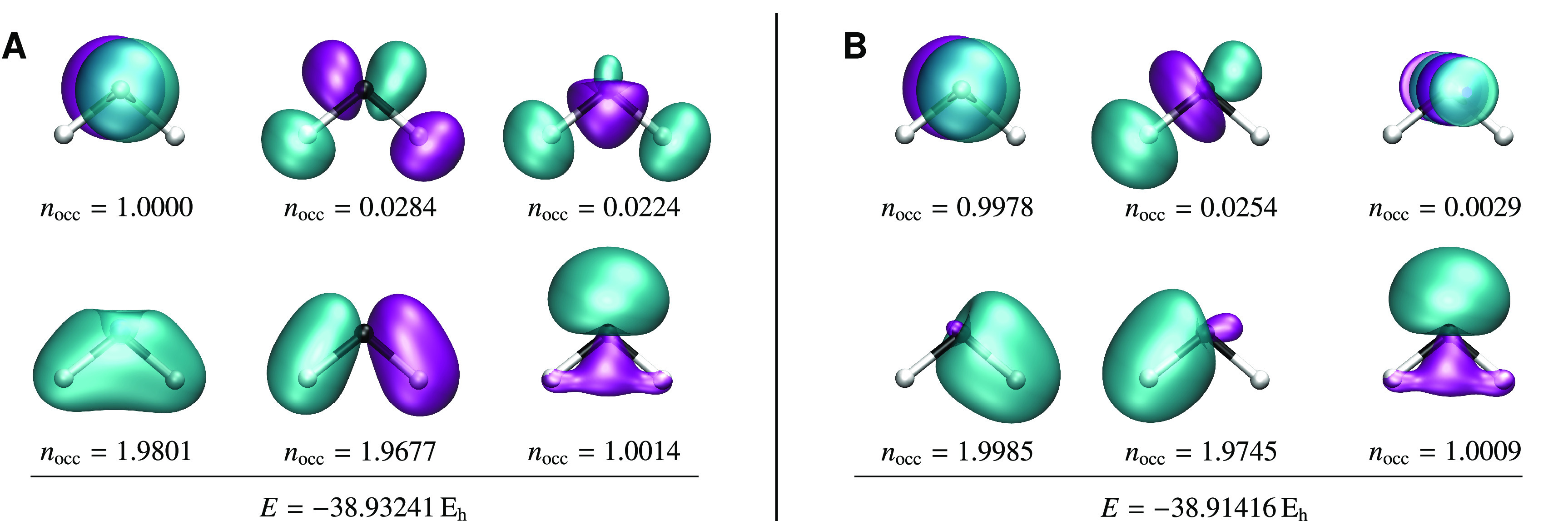
Comparison of the active orbitals for the two lowest-energy triplet CASSCF solutions for CH_2_ (6-31G) using the full valence (6,6) active space at a bond angle of 102°. (A) The active orbitals for the local minimum represent the chemically intuitive valence space. (B) For the unphysical index-1 saddle point, one of the antibonding C–H σ* orbitals with *n*_occ_ ≈ 0 is replaced by a carbon 3p orbital with *n*_occ_ = 0.0029. The remaining σ and σ* orbitals localize onto the C–H bonds.

While the larger active space alleviates root flipping, it also causes more unphysical solutions associated with redundant active orbitals. For example, the triplet ground state (dominated by two configurations) is represented by one SS-CASSCF(2,2) solution, but there are several higher-energy solutions in the (6,6) active space. Analogously to the H_2_ ground state, these additional solutions have higher Hessian indexes, with two index-1 and one index-2 saddle points represented in Figure 5. Again, the main difference from the true ground state is the active orbitals with occupation numbers close to zero, as illustrated for the global minimum and lowest-energy index-1 saddle point in Figure 6. Furthermore, we find an additional local minimum and index-1 saddle point that represent the ^1^A_1_ state. While all the triplet solutions give approximately the same equilibrium bond angle, the unphysical stationary points shift the crossing point to coincide with the singlet equilibrium geometry. This qualitative change in the energy surface would create a near-barrierless decay from the singlet excited state to the triplet ground state, demonstrating the importance of verifying the physicality of state-specific solutions.

### 3.3 Avoided Crossing in Lithium Fluoride

#### 3.3.1 Physicality of Multiple Solutions

The LiF binding curve provides a typical example of an avoided crossing. The ground state has ionic character at equilibrium but becomes a covalent state with almost no dipole moment in the dissociation limit. Multiple HF solutions are known to behave “quasi-diabatically” and cross each other at the physical avoided crossing.^47,143^ On the other hand, Bauschlicher and Langhoff demonstrated that this avoided crossing can lead to discontinuities in the CASSCF ground- and excited-state energy surfaces.^144^ Here we start by considering the state-specific singlet CASSCF solutions in the 6-31G basis set.

**Figure 7:**
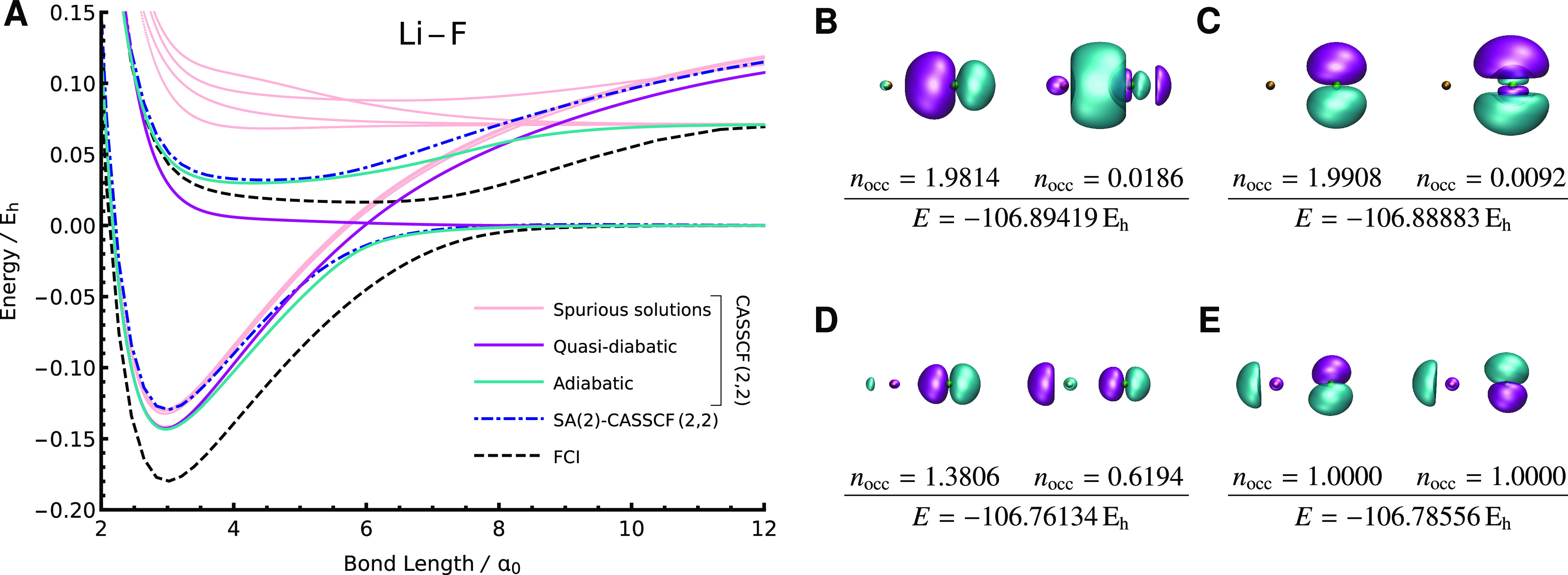
(A) The SS-CASSCF(2,2) approach gives many solutions for the LiF binding curve (6-31G) when the ground or excited state is dominated by a single configuration. Ground- and excited-state solutions with a suitable active space (B, D) behave adiabatically at the avoided crossing (cyan lines). Additional solutions with unsuitable active orbitals can represent either the ionic equilibrium configuration (C) or the covalent dissociation configuration (E) and behave quasi-diabatically at the avoided crossing (purple lines). The active orbitals are plotted at *R*(Li–F) = 4 *a*_0_. Exact FCI and SA(2)-CASSCF(2,2) data are taken from ref (47).

Using the minimal (2,2) active space, we search for stationary points with Hessian indices of 0 to 10 at *R*(Li–F) = 2.75 *a*_0_ (near the equilibrium geometry) using 1000 random starting points for each index. The active space for the SS-CASSCF global minimum contains the valence bonding σ and antibonding σ* orbitals with occupation numbers close to 2 and 0, respectively (Figure 7B). Because the exact wave function is dominated by a single closed-shell configuration, there are many additional solutions that are close to the ground-state energy at the equilibrium geometry. For example, the second-lowest-energy solution has an active space containing the out-of-plane fluorine 2p_*x*/*y*_ and 3p_*x*/*y*_ orbitals with occupation numbers close to 2 and 0, respectively (Figure 7C). This active space accounts for the radial correlation on the fluorine atom, providing a more balanced description of F and F^–^.^144^ In contrast, the exact excited state is more multiconfigurational at short bond lengths and is accurately represented by only one solution (Figure 7D), alongside a spurious symmetry-broken solution with diradical character (Figure 7E). These characteristics are reversed for bond lengths longer than the avoided crossing, where the excited state has closed-shell character with a large number of solutions and the ground state is represented by only two solutions.

State-specific CASSCF solutions can behave both quasi-diabatically and adiabatically in the vicinity of the avoided crossing. As the bond length changes, the unphysical solutions do not have the correct active orbitals to capture the strong correlation at the avoided crossing. Therefore, the two lowest-energy unphysical solutions intersect quasi-diabatically (dark purple in Figure 7A, corresponding to the solutions in Figure 7C and 7E). On the other hand, the physically meaningful solutions behave adiabatically and correctly avoid each other (cyan in Figure 7A). In principle, a linear expansion of both the quasi-diabatic and adiabatic states may provide a more accurate representation of the avoided crossing by introducing some of the dynamic correlation captured by the unphysical solutions. This expansion would require a multiconfigurational variant of nonorthogonal CI,^143^ where the Hamiltonian and overlap matrix elements can be efficiently computed using the nonorthogonal framework developed in refs (102) and (103).

While a complete description of the avoided crossing requires dynamic correlation,^145^ the advantage of state-specific orbital relaxation is still clear in the dissociation limit. The physical SS-CASSCF excitation energy tends toward the exact FCI result for the separated Li^+^···F^–^ configuration, while state-averaged calculations (with an equal weighting for the two states) provide an overestimate (Figure 7A). In this SS-CASSCF solution, the σ and σ* orbitals (Figure 7A) both localize to give 2p_*z*_ orbitals that accurately represent the F^–^ anion. Consequently, as expected, the state-specific formalism provides a more accurate representation of this charge transfer excitation than a state-averaged approach.

#### 3.3.2 Elucidating the Bauschlicher–Langhoff Discontinuity

The seminal CASSCF investigation of LiF, by Bauschlicher and Langhoff, highlighted the presence of a discontinuity in the ground-state dipole moment in the vicinty of the avoided crossing.^144^ This discontinuity is a signature of a discontinuity in the wave function, which manifests as a cusp in the corresponding energy surface. This phenomenon, which we name the “Bauschlicher–Langhoff discontinuity”, has long been used as key evidence for the potential issues of state-specific calculations in the vicinity of an avoided crossing. Malrieu and co-workers attributed its origin to a near degeneracy between the closed-shell ionic and open-shell covalent configurations and described a lower-energy covalent state that emerges from a potential symmetry-breaking point as the bond length increases.^119^ The framework developed here and the advance in computing over the past 30 years now allow this topological characterization to be rigorously tested.

**Figure 8:**
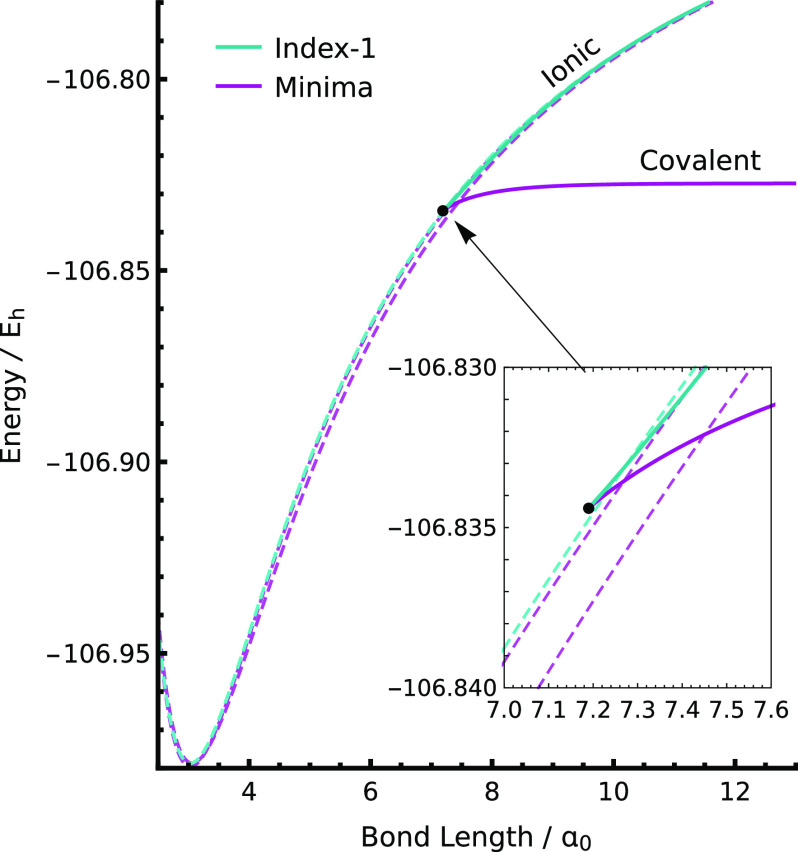
Topology of the low-energy SS-CASSCF(2, 2) solutions near the Bausclicher–Langhoff discontinuity in LiF^119,144^ using the basis set defined in ref (144). A cusp in the ground-state energy occurs when two local minima cross, while the covalent structure coalesces with an index-1 saddle point at a pair annihilation point (black dot).

To identify the relevant solutions, we searched for minima and index-1 saddle points at a bond length of 8.50 *a*_0_ using 1000 random starting points, a (2,2) active space, and the original basis set described in ref (144). At *R*(Li–F) = 8.5 *a*_0_, the global minimum corresponds to the covalent structure identified in ref (119). In addition, two local minima and two index-1 saddle points exist at higher energies, representing the ionic configurations (Figure 8). As the bond length is shortened, there is a crossing between the lowest-energy ionic and covalent minima near *R*(Li–F) = 7.4 *a*_0_, which we believe corresponds to the previously described discontinuity.^119,144^

Topologically, two nondegenerate minima cannot coalesce without the presence of an index-1 saddle point, and thus, the disappearance described by Malrieu and co-workers cannot be the full picture.^119^ Instead, we find that the covalent structure crosses the two lowest-energy local minima and eventually coalesces with an index-1 saddle point representing the ionic configurations. Following the downhill directions from this index-1 saddle point reveals that it connects the covalent local minimum with the lowest-energy ionic local minimum. Furthermore, the downhill Hessian eigenvector has significant orbital and CI components, which highlights the strong coupling between the different degrees of freedom in the vicinity of the avoided crossing. Both solutions disappear at this point (black dot in Figure 8), and thus, there is no quasi-diabatic covalent solution at shorter bond lengths.

In the mathematical framework of catastrophe theory,^146^ this type of coalescence can be classified as a fold catastrophe or a pair annihilation point. Singularities in this class have previously been identified and characterized for multiple HF solutions,^122^ where they most commonly occur in asymmetric molecules, for example LiF,^47,143^ H–Z^40^ (for a partial nuclear charge *Z*), and ethylene analogues.^40^ The discontinuous jump in the energy at the pair annihilation point in LiF will create issues for calculations that attempt to follow the covalent solution across multiple bond lengths, making these solutions unsuitable for techniques such as *ab initio* molecular dynamics. Furthermore, since the lowest-energy covalent and ionic local minima cross rather than coalesce, the gradient of the global-minimum energy at the crossing point is discontinuous and there is an unphysical cusp in the resulting energy surface.

The absence of this pair annihilation point using 6-31G compared to Bauschlicher and Langhoff’s basis set demonstrates how the topology of multiple CASSCF solutions can be affected by the AO basis. We suspect that these differences arise from the subtle changes in the underlying energy landscape that affect the relative stability of different solutions. However, these results demonstrate the danger of generalizing conclusions from one basis set to another, even for the same molecule.

## 4 Conclusions

State-specific approximations promise to provide a more balanced representation of electronic excitations by independently optimizing both the ground- and excited-state wave functions. In this work, we have investigated the energy landscape for excited state-specific stationary points in the multiconfigurational CASSCF approach. We have shown how state-specific approximations can accurately describe high-energy and charge transfer excitations, beyond the reach of state-averaged calculations with small active spaces. However, the CASSCF energy landscape can have a large number of stationary points, which complicates the selection and interpretation of physically relevant solutions.

Multiple stationary points in state-specific CASSCF calculations arise through two primary mechanisms. First, many solutions occur when the active space is too large for the static correlation that must be described. In this case, the redundant active orbitals with *n*_occ_ ≈ 0 can be interchanged with virtual orbitals without significantly changing the energy, creating a series of stationary points with an increasing number of downhill Hessian eigendirections. Active orbitals with *n*_occ_ ≈ 2 can be interchanged with doubly occupied inactive orbitals in a similar fashion. The number of these solutions grows as the AO basis set gets larger. On the other hand, symmetry-broken solutions occur when the active space is too small to describe the static correlation mechanisms, causing the CASSCF wave function to become “pinned” onto a subset of the configurations in the exact wave function. These results demonstrate the importance of finding a “Goldilocks region” where the active space is neither too large or too small but just right.

Unphysical solutions can have important consequences for the resulting potential energy surfaces. For example, while choosing the wrong active space only introduces a small energy error when the wave function is dominated by a single closed-shell configuration, it can prevent the CASSCF wave function from correctly capturing static correlation when the molecular structure changes. The active space for stationary points does not change significantly along a reaction coordinate, meaning that the incorrect active orbitals remain for all geometries. For ground-state calculations, one can rely on following downhill directions away from saddle points to obtain a more suitable local minimum, hopefully with the best active space. However, it is hard to predict which Hessian index will give the most physical stationary point for an excited state, and thus, choosing the most accurate excited-state stationary point is challenging without prior chemical intuition. It has long been known that the right choice of active orbitals is key to the success of CASSCF, but the current results demonstrate the severity of this challenge for state-specific excitations.

In addition, we have investigated the topology of SS-CASSCF(2,2) solutions near the singlet–triplet crossing in CH_2_ and the covalent–ionic avoided crossing in LiF. We observe unphysical root flipping where the CH_2_ excited-state solution is a local minimum near the crossing point before becoming an index-1 saddle point further along the reaction trajectory. This phenomenon occurs because the state-specific orbital optimization artificially stabilizes the local minima, and this is still present in the full-valence (6,6) active space. Furthermore, the change in Hessian index is associated with an additional spin-contaminated index-1 saddle point that connects the singlet and triplet stationary points. The presence of zero Hessian eigenvalues at these instability thresholds may cause numerical issues for second-order optimization algorithms. On the other hand, for the LiF avoided crossing, we have observed the coalescence of the local covalent minimum with an index-1 saddle point representing the ionic state, which both disappear entirely at shorter bond lengths. While this pairwise coalescence depends on the basis set, it would catastrophically affect the applicability of SS-CASSCF for generating smooth and continuous potential energy surfaces.

Moving forward, SS-CASSCF calculations must overcome the troublesome issues of multiple solutions. Practical solutions may rely on the identification of suitable initial guesses from more black-box techniques or by focusing on optimization algorithms that target desirable excited-state physical properties (e.g., dipole moments), such as the generalized variational principles developed by Hanscam and Neuscamman.^91^ Alternatively, more bespoke excited-state wave function *ansätze*, such as minimal configuration state functions^69^ or excited-state mean-field theory,^64,65,68^ may remove unphysical solutions associated with redundant active orbitals and avoid the disappearance of solutions at pairwise coalescence points. Surmounting these issues will allow the benefits of state-specific calculations for computing excited states with bespoke orbitals and small active spaces to be fully realized.

## Supporting Information Available

Derivation of the gradient and second derivatives for the CASSCF energy and description of eigenvector-following and Newton–Raphson optimization algorithms used (PDF)

## References

[ref1] Burton H. G. A. Energy Landscape of State-Specific Electronic Structure Theory. J. Chem. Theory Comput. 2022, 18, 1512 10.1021/acs.jctc.1c01089. 35179023 PMC9082508

[ref2] Olsen J.; Jørgensen P.; Yeager D. L. Multiconfigurational Hartree–Fock studies of avoided curve crossing using the Newton–Raphson technique. J. Chem. Phys. 1982, 76, 527 10.1063/1.442699.

[ref3] Golab J. T.; Yeager D. L.; Jørgensen P. Proper characterization of MC SCF stationary points. Chem. Phys. 1983, 78, 175 10.1016/0301-0104(83)85106-4.

[ref4] Olsen J.; Yeager D. L.; Jørgensen P. Optimization and Characterization of a Multiconfigurational Self-Consistent Field (MCSCF) State. Adv. Chem. Phys. 2007, 54, 1 10.1002/9780470142783.ch1.

[ref5] Golab J. T.; Yeager D. L.; Jørgensen P. Multiple stationary point representations in MC SCF calculations. Chem. Phys. 1985, 93, 83 10.1016/0301-0104(85)85051-5.

[ref6] Bacalis N. C.; Xiong Z.; Zang J.; Karaoulanis D. Computing correct truncated excited state wavefunctions. AIP Conf. Proc. 2016, 1790, 020007 10.1063/1.4968633.

[ref7] Bacalis N. C. If Truncated Wave Functions of Excited State Energy Saddle Points Are Computed as Energy Minima, Where Is the Saddle Point? In Theoretical Chemistry for Advanced Nanomaterials: Functional Analysis by Computation and Experiment; Onishi T., Ed.; Springer: Singapore, 2020; p 465.

[ref8] Runge E.; Gross E. K. U. Density-Functional Theory for Time-Dependent Systems. Phys. Rev. Lett. 1984, 52, 997 10.1103/PhysRevLett.52.997.

[ref9] Dreuw A.; Head-Gordon M. Single-Reference ab Initio Methods for the Calculation of Excited States of Large Molecules. Chem. Rev. 2005, 105, 4009 10.1021/cr0505627. 16277369

[ref10] Burke K.; Werschnik J.; Gross E. K. U. Time-dependent density functional theory: Past and future. J. Chem. Phys. 2005, 123, 062206 10.1063/1.1904586. 16122292

[ref11] Hait D.; Rettig A.; Head-Gordon M. Beyond the Coulson-Fischer point: characterizing single excitation CI and TDDFT for excited states in single bond dissociations. Phys. Chem. Chem. Phys. 2019, 21, 21761 10.1039/C9CP04452C. 31552963

[ref12] Maitra N. T.; Zhang F.; Cave R. J.; Burke K. Double excitations within time-dependent density functional theory linear response. J. Chem. Phys. 2004, 120, 5932 10.1063/1.1651060. 15267474

[ref13] Schirmer J. Beyond the random-phase approximation: A new approximation scheme for the polarization propagator. Phys. Rev. A 1982, 26, 2395 10.1103/PhysRevA.26.2395.

[ref14] Dreuw A.; Wormit M. The algebraic diagrammatic construction scheme for the polarization propagator for the calculation of excited states. Wiley Interdiscip. Rev.: Comput. Mol. Sci. 2015, 5, 82 10.1002/wcms.1206.

[ref15] Stanton J. F.; Bartlett R. J. The equation of motion coupled-cluster method. A systematic biorthogonal approach to molecular excitation energies, transition probabilities, and excited state properties. J. Chem. Phys. 1993, 98, 7029 10.1063/1.464746.

[ref16] Krylov A. I. Equation-of-Motion Coupled-Cluster Methods for Open-Shell and Electronically Excited Species: The Hitchhiker’s Guide to Fock Space. Annu. Rev. Phys. Chem. 2008, 59, 433 10.1146/annurev.physchem.59.032607.093602. 18173379

[ref17] Tozer D. J. Relationship between Long-Range Charge-Transfer Excitation Energy Error and Integer Discontinuity in Kohn–Sham Theory. J. Chem. Phys. 2003, 119, 12697 10.1063/1.1633756.

[ref18] Dreuw A.; Head-Gordon M. Failure of Time-Dependent Density Functional Theory for Long-Range Charge-Transfer Excited States: The Zincbateriochlorin–Bacteriochloring and Bacteriochlorophyll—Spheroidene Complexes. J. Am. Chem. Soc. 2004, 126, 4007 10.1021/ja039556n. 15038755

[ref19] McLachlan A. D.; Ball M. A. Time-Dependent Hartree–Fock Theory for Molecules. Rev. Mod. Phys. 1964, 36, 844 10.1103/RevModPhys.36.844.

[ref20] Bartlett R. J. Coupled-cluster theory and its equation-of-motion extensions. Wiley Interdiscip. Rev.: Comput. Mol. Sci. 2012, 2, 126 10.1002/wcms.76.

[ref21] Helmich-Paris B. Benchmarks for Electronically Excited States with CASSCF methods. J. Chem. Theory Comput. 2019, 15, 4170 10.1021/acs.jctc.9b00325. 31136706 PMC6620717

[ref22] Gilbert A. T. B.; Besley N. A.; Gill P. M. W. Self-Consistent Field Calculations of Excited States Using the Maximum Overlap Method (MOM). J. Phys. Chem. A 2008, 112, 13164 10.1021/jp801738f. 18729344

[ref23] Barca G. M. J.; Gilbert A. T. B.; Gill P. M. W. Communication: Hartree–Fock description of excited states of H_2_. J. Chem. Phys. 2014, 141, 111104 10.1063/1.4896182. 25240338

[ref24] Barca G. M. J.; Gilbert A. T. B.; Gill P. M. W. Simple Models for Difficult Electronic Excitations. J. Chem. Theory Comput. 2018, 14, 1501 10.1021/acs.jctc.7b00994. 29444408

[ref25] Hait D.; Head-Gordon M. Orbital Optimized Density Functional Theory for Electronic Excited States. J. Phys. Chem. Lett. 2021, 12, 4517 10.1021/acs.jpclett.1c00744. 33961437

[ref26] Hait D.; Head-Gordon M. Excited State Orbital Optimization via Minimizing the Square of the Gradient: General Approach and Application to Singly and Doubly Excited States via Density Functional Theory. J. Chem. Theory Comput. 2020, 16, 1699 10.1021/acs.jctc.9b01127. 32017554

[ref27] Carter-Fenk K.; Herbert J. M. State-Targeted Energy Projection: A Simple and Robust Approach to Orbital Relaxation of Non-Aufbau Self-Consistent Field Solutions. J. Chem. Theory Comput. 2020, 16, 5067 10.1021/acs.jctc.0c00502. 32644792

[ref28] Levi G.; Ivanov A. V.; Jónsson H. Variational Density Functional Calculations of Excited States via Direct Optimization. J. Chem. Theory Comput. 2020, 16, 6968 10.1021/acs.jctc.0c00597. 33064484

[ref29] Levi G.; Ivanov A. V.; Jónsson H. Variational calculations of excited states via direct optimization of the orbitals in DFT. Faraday Discuss. 2020, 224, 448 10.1039/D0FD00064G. 32935688

[ref30] Ivanov A. V.; Levi G.; Jónsson E. Ö.; Jónsson H. Method for Calculating Excited Electronic States Using Density Functionals and Direct Orbital Optimization with Real Space Grid or Plane-Wave Basis Set. J. Chem. Theory Comput. 2021, 17, 5034 10.1021/acs.jctc.1c00157. 34227810

[ref31] Shea J. A. R.; Neuscamman E. Size Consistent Excited States via Algorithmic Transformations between Variational Principles. J. Chem. Theory Comput. 2017, 13, 6078 10.1021/acs.jctc.7b00923. 29140699

[ref32] Jankowski K.; Kowalski K.; Jankowski P. Applicability of single-reference coupled-cluster methods to excited states. A model study. Chem. Phys. Lett. 1994, 222, 608 10.1016/0009-2614(94)00391-2.

[ref33] Jankowski K.; Kowalski K.; Jankowski P. Multiple Solutions of the Single-Reference Coupled-Cluster Equations. II. Alternative Reference States. Int. J. Quantum Chem. 1995, 53, 501 10.1002/qua.560530507.

[ref34] Piecuch P.; Kowalski K. In Search of the Relationship between Multiple Solutions Characterizing Coupled-Cluster Theories. In Computational Chemistry: Reviews of Current Trends; Leszczynski J., Ed.; World Scientific, 2000; Vol. 5, Chapter 1, p 1.

[ref35] Mayhall N. J.; Raghavachari K. Multiple Solutions to the Single-Reference CCSD equations for NiH. J. Chem. Theory Comput. 2010, 6, 2714 10.1021/ct100321k. 26616072

[ref36] Lee J.; Head-Gordon M. Distinguishing artifical and essential symmetry breaking in a single determinant: approach and application to the C_60_, C_36_ and C_20_ fullerenes. Phys. Chem. Chem. Phys. 2019, 21, 4763 10.1039/C8CP07613H. 30762069

[ref37] Kossoski F.; Marie A.; Scemama A.; Caffarel M.; Loos P.-F. Excited States from State-Specific Orbital-Optimized Pair Coupled Cluster. J. Chem. Theory Comput. 2021, 17, 4756 10.1021/acs.jctc.1c00348. 34310140 PMC8359009

[ref38] Marie A.; Kossoski F.; Loos P.-F. Variational coupled cluster for ground and excited states. J. Chem. Phys. 2021, 155, 104105 10.1063/5.0060698. 34525834

[ref39] Burton H. G. A.; Wales D. J. Energy Landscapes for Electronic Structure. J. Chem. Theory Comput. 2021, 17, 151 10.1021/acs.jctc.0c00772. 33369396

[ref40] Burton H. G. A.; Gross M.; Thom A. J. W. Holomorphic Hartree–Fock Theory: The Nature of Two-Electron Problems. J. Chem. Theory Comput. 2018, 14, 607 10.1021/acs.jctc.7b00980. 29320177

[ref41] Coulson C. A.; Fischer I. XXXIV. Notes on the molecular orbital treatment of the hydrogen molecule. Philos. Mag. 1949, 40, 386 10.1080/14786444908521726.

[ref42] Fukutome H. The Unrestricted Hartree–Fock Theory of Chemical Reactions. III: Instability Conditions for Paramagnetic and Spin Density Wave States and Application to Internal Rotation of Ethylene. Prog. Theor. Phys. 1973, 50, 1433 10.1143/PTP.50.1433.

[ref43] Fukutome H. Theory of the Unrestricted Hartree–Fock equation and Its Solutions. III: Classification of Instabilities and Interconnection Relation between the Eight Classes of UHF Solutions. Prog. Theor. Phys. 1974, 52, 1766 10.1143/PTP.52.1766.

[ref44] Fukutome H. Theory of the Unrestricted Hartree–Fock equation and Its Solutions III: Classification and Characterization of UHF Solutions by Their Behaviour for Spin Rotation and Time Reversal. Prog. Theor. Phys. 1974, 52, 115 10.1143/PTP.52.115.

[ref45] Ye H.-Z.; Welborn M.; Ricke N. D.; Van Voorhis T. σ-SCF: A direct energy-targeting method to mean-field excited states. J. Chem. Phys. 2017, 147, 214104 10.1063/1.5001262. 29221390

[ref46] Thom A. J. W.; Head-Gordon M. Locating Multiple Self-Consistent Field Solutions: An Approach Inspired by Metadynamics. Phys. Rev. Lett. 2008, 101, 193001 10.1103/PhysRevLett.101.193001. 19113263

[ref47] Burton H. G. A.; Thom A. J. W. Reaching Full Correlation through Nonorthogonal Configuration Interaction: A Second-Order Perturbative Approach. J. Chem. Theory Comput. 2020, 16, 5586 10.1021/acs.jctc.0c00468. 32786910

[ref48] Jensen K. T.; Benson R. L.; Cardamone S.; Thom A. J. W. Modeling Electron Transfers Using Quasidiabatic Hartree–Fock States. J. Chem. Theory Comput. 2018, 14, 4629 10.1021/acs.jctc.8b00379. 30060649

[ref49] Vaucher A. C.; Reiher M. Steering Orbital Optimization out of Local Minima and Saddle Points toward Lower Energy. J. Chem. Theory Comput. 2017, 13, 1219 10.1021/acs.jctc.7b00011. 28207264

[ref50] Dong X.; Mahler A. D.; Kempfer-Robertson E. M.; Thompson L. M. Global Elucidation of Self-Consistent Field Solution Space Using Basin Hopping. J. Chem. Theory Comput. 2020, 16, 5635 10.1021/acs.jctc.0c00488. 32787181

[ref51] Szabo A.; Ostlund N. S. Modern Quantum Chemistry; Dover Publications: New York, 1989.

[ref52] Das G.; Wahl A. C. Extended Hartree—Fock Wavefunctions: Optimized Valence Configurations for H_2_ and Li_2_, Optimized Double Configurations for F_2_. J. Chem. Phys. 1966, 44, 87 10.1063/1.1726508.

[ref53] Roos B. O.; Taylor P. R.; Sigbahn P. E. M. A complete active space SCF method (CASSCF) using a density matrix formulated super-CI. Chem. Phys. 1980, 48, 157 10.1016/0301-0104(80)80045-0.

[ref54] Roos B. O. The Complete Active Space SCF method in a Fock-Matrix-Based Super-CI Formulation. Int. J. Quantum Chem. 1980, 18, 175 10.1002/qua.560180822.

[ref55] Roos B. O.; Lindh R.; Malmqvist P. Å; Veryazov V.; Windmark P.-O. Multiconfigurational Quantum Chemistry; Wiley, 2016.

[ref56] Das G. Multiconfiguration self-consistent field (MCSCF) theory for excited states. J. Chem. Phys. 1973, 58, 5104 10.1063/1.1679100.

[ref57] Krauss M.; Neumann D. B. The ^5^Σ_g_^+^ states of N_2_. Mol. Phys. 1976, 32, 101 10.1080/00268977600101641.

[ref58] Bauschlicher C. W. Jr.; Yarkony D. R. Electronic structure of CaO. I. J. Chem. Phys. 1978, 68, 3990 10.1063/1.436312.

[ref59] Bauschlicher C. W. Jr.; Yarkony D. R. MCSCF wave functions for excited states of polar moleculars: Application to BeO. J. Chem. Phys. 1980, 72, 1138 10.1063/1.439255.

[ref60] Bauschlicher C. W. Jr.; Lengsfield B. H. III; Yarkony D. R. On the low lying singlet states of BeO. J. Chem. Phys. 1980, 73, 5702 10.1063/1.440048.

[ref61] Bauschlicher C. W. Jr.; Silver D. M.; Yarkony D. R. An SCF and and MCSCF description of the low-lying states of MgO. J. Chem. Phys. 1980, 73, 2867 10.1063/1.440456.

[ref62] Guihery N.; Malrieu J.-P.; Maynau D.; Handrick K. Unexpected CASSCF Bistability Phenomenon. Int. J. Quantum Chem. 1997, 61, 45 10.1002/(SICI)1097-461X(1997)61:1<45::AID-QUA5>3.0.CO;2-4.

[ref63] Angeli C.; Calzado C. J.; Cimiraglia R.; Evangelisti S.; Mayna D. Multiple complete active space self-consistent field solutions. Mol. Phys. 2003, 101, 1937 10.1080/0026897031000109293.

[ref64] Shea J. A. R.; Neuscamman E. A mean field platform for excited state quantum chemistry. J. Chem. Phys. 2018, 149, 081101 10.1063/1.5045056. 30193500

[ref65] Shea J. A. R.; Gwin E.; Neuscamman E. A Generalized Variational Principle with Applications to Excited State Mean Field Theory. J. Chem. Theory Comput. 2020, 16, 1526 10.1021/acs.jctc.9b01105. 32017562

[ref66] Zhao L.; Neuscamman E. Excited state mean-field theory without automatic differentiation. J. Chem. Phys. 2020, 152, 204112 10.1063/5.0003438. 32486659

[ref67] Zhao L.; Neuscamman E. Density Functional Extension to Excited-State Mean-Field Theory. J. Chem. Theory Comput. 2020, 16, 164 10.1021/acs.jctc.9b00530. 31765142

[ref68] Hardikar T. S.; Neuscamman E. A self-consistent field formulation of excited state mean field theory. J. Chem. Phys. 2020, 153, 164108 10.1063/5.0019557. 33138440

[ref69] Kossoski F.; Loos P.-F. State-Specific Configuration Interaction for Excited States. J. Chem. Theory Comput. 2023, 19, 2258 10.1021/acs.jctc.3c00057. 37024102 PMC10134430

[ref70] Dalgaard E.; Jørgensen P. Optimization of orbitals for multiconfigurational reference states. J. Chem. Phys. 1978, 69, 3833 10.1063/1.437049.

[ref71] Dalgaard E. A quadratically convergent reference state optimization procedure. Chem. Phys. Lett. 1979, 65, 559 10.1016/0009-2614(79)80291-2.

[ref72] Yeager D. L.; Jørgensen P. Convergency studies of second and approximate second order multiconfigurational Hartree–Fock procedures. J. Chem. Phys. 1979, 71, 755 10.1063/1.438363.

[ref73] Lengsfield B. H. III General second order MCSCF theory: A density matrix directed algorithm. J. Chem. Phys. 1980, 73, 382 10.1063/1.439885.

[ref74] Siegbahn P. E. M.; Almlöf J.; Heiberg A.; Roos B. O. The complete active space SCF (CASSCF) method in a Newton-Raphson formulation with application to the HNO molecule. J. Chem. Phys. 1981, 74, 2384 10.1063/1.441359.

[ref75] Werner H.-J.; Meyer W. A quadratically convergent MCSCF method for the simultaneous optimization of several states simultaneous optimization of several states. J. Chem. Phys. 1981, 74, 5794 10.1063/1.440892.

[ref76] Werner H.-J.; Knowles P. J. A second order multiconfigurational SCF procedure with optimum convergence. J. Chem. Phys. 1985, 82, 5053 10.1063/1.448627.

[ref77] Yeager D. L.; Jørgensen P. A numerical study of the convergency of second and approximate second-order multiconfiguration Hartree–Fock procedures. Mol. Phys. 1980, 39, 587 10.1080/00268978000100491.

[ref78] Yeager D. L.; Albertsen P.; Jørgensen P. Mode damping in multiconfigurational Hartree–Fock procedures. J. Chem. Phys. 1980, 73, 2811 10.1063/1.440450.

[ref79] Jørgensen P.; Olsen J.; Yeager D. L. Generalizations of Newton–Raphson and multiplicity independent Newton–Raphson approaches in multiconfigurational Hartree–Fock theory. J. Chem. Phys. 1981, 75, 5802 10.1063/1.442029.

[ref80] Yeager D. L.; Lynch D.; Nichols J.; Jørgensen P.; Olsen J. Newton–Raphson Approaches and Generalizations in Multiconfigurational Self-Consistent Field Calculations. J. Phys. Chem. 1982, 86, 2140 10.1021/j100209a006.

[ref81] Sun Q.; Yang J.; Chan G. K.-L. A General Second Order Complete Active Space Self-Consistent-Field Solver for Large-Scale Systems. Chem. Phys. Lett. 2017, 683, 291 10.1016/j.cplett.2017.03.004.

[ref82] Kreplin D. A.; Knowles P. J.; Werner H.-J. Second-order MCSCF optimization revisited. I. Improved algorithms for fast and robust second-order CASSCF convergence. J. Chem. Phys. 2019, 150, 194106 10.1063/1.5094644. 31117783

[ref83] Kreplin D. A.; Knowles P. J.; Werner H.-J. MCSCF optimization revisited. II. Combined first- and second-order orbital optimization for large molecules. J. Chem. Phys. 2020, 152, 074102 10.1063/1.5142241. 32087666

[ref84] Rizzo A.; Yeager D. L. Characteristics and some peculiarities of multi-configurational self-consistent field stationary points of the Li^–^ ground state. J. Chem. Phys. 1990, 93, 8011 10.1063/1.459330.

[ref85] Zaitsevskii A.; Malrieu J.-P. The discontinuities of state-average MCSCF potential surfaces. Chem. Phys. Lett. 1994, 228, 458 10.1016/0009-2614(94)00899-X.

[ref86] Yeager D. L.; Jørgensen P. A Multiconfigurational Time-Dependent Hartree–Fock Approach. Chem. Phys. Lett. 1979, 65, 77 10.1016/0009-2614(79)80130-X.

[ref87] Dalgaard E. Time-dependent Multiconfigurational Hartree–Fock Theory. J. Chem. Phys. 1980, 72, 816 10.1063/1.439233.

[ref88] Olsen J.; Jørgensen P. Linear and Nonlinear Response Functions for an Exact State and for an MCSCF State. J. Chem. Phys. 1985, 82, 3235 10.1063/1.448223.

[ref89] Helmich-Paris B. CASSCF Linear Response Calculations for Large Open-Shell Molecules. J. Chem. Phys. 2019, 150, 174121 10.1063/1.5092613. 31067879

[ref90] Tran L. N.; Neuscamman E. Improving Excited-State Potential Energy Surfaces via Optimal Orbital Shapes. J. Phys. Chem. A 2020, 124, 8273 10.1021/acs.jpca.0c07593. 32885970

[ref91] Hanscam R.; Neuscamman E. Applying Generalized Variational Principles to Excited-State-Specific Complete Active Space Self-consistent Field Theory. J. Chem. Theory Comput. 2022, 18, 6608 10.1021/acs.jctc.2c00639. 36215108

[ref92] Tran L. N.; Shea J. A. R.; Neuscamman E. Tracking Excited States in Wave Function Optimization Using Density Matrices and Variational Principles. J. Chem. Theory Comput. 2019, 15, 4790 10.1021/acs.jctc.9b00351. 31393725

[ref93] Helgaker T.; Jørgensen P.; Olsen J. Molecular Electronic-Structure Theory; John Wiley & Sons: Chichester, U.K., 2000.

[ref94] Knowles P. J.; Handy N. C. A new determinant-based full configuration interaction method. Chem. Phys. Lett. 1984, 111, 315 10.1016/0009-2614(84)85513-X.

[ref95] Olsen J.; Jørgensen P.; Simons J. Passing the one-billion limit in full configuration-interaction (FCI) calculations. Chem. Phys. Lett. 1990, 169, 463 10.1016/0009-2614(90)85633-N.

[ref96] Siegbahn P. E. M. A new direct CI method for large CI expansions in a small orbital space. Chem. Phys. Lett. 1984, 109, 417 10.1016/0009-2614(84)80336-X.

[ref97] Head-Gordon M.; Maslen P. E.; White C. A. A tensor formulation of many-electron theory iin a nonorthogonal single-particle basis. J. Chem. Phys. 1998, 108, 616 10.1063/1.475423.

[ref98] Hylleraas E. A.; Undheim B. Numerische Berechnung der 2S-Terme von Ortho- und Par-Helium. Z. Phys. 1930, 65, 759 10.1007/BF01397263.

[ref99] MacDonald J. K. L. Successive Approximations by the Rayleigh-Ritz Variation Method. Phys. Rev. 1933, 43, 830 10.1103/PhysRev.43.830.

[ref100] Douady J.; Ellinger Y.; Subra R.; Levy B. Exponential transformation of molecular orbitals: A quadratically convergent SCF procedure. I. General formulation and application to closed-shell ground states. J. Chem. Phys. 1980, 72, 1452 10.1063/1.439369.

[ref101] Van Voorhis T.; Head-Gordon M. A geometric approach to direct minimization. Mol. Phys. 2002, 100, 1713 10.1080/00268970110103642.

[ref102] Burton H. G. A. Generalized nonorthogonal matrix elements: Unifying Wick’s theorem and the Slater–Condon rules. J. Chem. Phys. 2021, 154, 144109 10.1063/5.0045442. 33858143

[ref103] Burton H. G. A. Generalized nonorthogonal matrix elements. II: Extension to arbitrary excitations. J. Chem. Phys. 2022, 157, 204109 10.1063/5.0122094. 36456247

[ref104] Mayer I. Simple Theorems, Proofs, and Derivations in Quantum Chemistry; Springer: New York, 2003.

[ref105] Löwdin P.-O. Quantum Theory of Many-Particle Systems. I. Physical Interpretations by Means of Density Matrices, Natural Spin-Orbitals, and Convergence Problems in the Method of Configurational Interaction. Phys. Rev. 1955, 97, 1474 10.1103/PhysRev.97.1474.

[ref106] Docken K. K.; Hinze J. LiH Potential Curves and Wavefunctions for X^1^Σ^+^, A^1^Σ^+^, B^1^Π, ^3^Σ^+^, and ^3^Π. J. Chem. Phys. 1972, 57, 4928 10.1063/1.1678164.

[ref107] Nottoli T.; Gauss J.; Lipparini F. Second-Order CASSCF algorithm with the Cholesky Decomposition of the Two-Electron Integrals. J. Chem. Theory Comput. 2021, 17, 6819 10.1021/acs.jctc.1c00327. 34719925 PMC8582256

[ref108] Helmich-Paris B. A trust-region augmented Hessian implementation for state-specific and state-averaged CASSCF wave functions. J. Chem. Phys. 2022, 156, 204104 10.1063/5.0090447. 35649854

[ref109] Cerjan C. J.; Miller W. H. On finding transition states. J. Chem. Phys. 1981, 75, 2800 10.1063/1.442352.

[ref110] Wales D. J. Structural and Topological Consequences of Anisotropic Interactions in Clusters. Faraday Discuss. 1990, 86, 3505 10.1039/ft9908603505.

[ref111] Wales D. J. Energy Landscapes: Applications to Clusters. Biomolecules and Glasses; Cambridge University Press: Cambridge, U.K., 2004.

[ref112] Hoffmann M. R.; Sherrill C. D.; Leininger M. L.; Schaefer H. F. III. Optimization of MCSCF excited states using directions of negative curvature. Chem. Phys. Lett. 2002, 355, 183 10.1016/S0009-2614(02)00208-7.

[ref113] Nocedal J.; Wright S. Numerical Optimization; Springer: New York, 2006.

[ref114] Jensen H. J. A.; Ågren H. A direct, restricted-step, second-order MC SCF program for large scale *ab initio* calculations. Chem. Phys. 1986, 104, 229 10.1016/0301-0104(86)80169-0.

[ref115] Sun Q.; Zhang X.; Banerjee S.; Bao P.; Barbry M.; Blunt N. S.; Bogdanov N. A.; Booth G. H.; Chen J.; Cui Z.-H.; et al. Recent developments in the PySCF program package. J. Chem. Phys. 2020, 153, 024109 10.1063/5.0006074. 32668948

[ref116] Humphrey W.; Dalke A.; Schulten K. VMD—Visual Molecular Dynamics. J. Mol. Graphics 1996, 14, 33 10.1016/0263-7855(96)00018-5. 8744570

[ref117] Mathematica, ver. 12.0.0; Wolfram Research, Inc.: Champaign, IL, 2021; https://www.wolfram.com/mathematica.

[ref118] Ditchfield R.; Hehre W. J.; Pople J. A. Self-Consistent Molecular-Orbital Methods. IX. An Extended Gaussian-Type Basis for Molecular-Orbital Studies of Organic Molecules. J. Chem. Phys. 1971, 54, 724 10.1063/1.1674902.

[ref119] Sanchez de Meras A.; Lepetit M.-B.; Malrieu J.-P. Discontinuity of valence CASSCF wave functions around weakly avoided crossing between valence configurations. Chem. Phys. Lett. 1990, 172, 163 10.1016/0009-2614(90)87291-X.

[ref120] Krishnan R.; Binkley J. S.; Seeger R.; Pople J. A. Self-consistent Molecular Orbital Methods. XX. A Basis Set for Correlated Wave Functions. J. Chem. Phys. 1980, 72, 650 10.1063/1.438955.

[ref121] Andersson K.; Roos B. O. Excitation energies in the nickel atom studied with the complete active space SCF method and second-order perturbation theory. Chem. Phys. Lett. 1992, 191, 507 10.1016/0009-2614(92)85581-T.

[ref122] Fukutome H. Theory of the Unrestricted Hartree–Fock equation and Its Solutions. IV: Behavior of UHF Solutions in the Vicinity of Interconnecting Instability Threshold. Prog. Theor. Phys. 1975, 53, 1320 10.1143/PTP.53.1320.

[ref123] Mestechkin M. M. Restricted Hartree–Fock Method Instability. Int. J. Quantum Chem. 1978, 13, 469 10.1002/qua.560130403.

[ref124] Mestechkin M. Instability Threshold and Peculiar Solutions of Hartree–Fock Equations. Int. J. Quantum Chem. 1979, 15, 601 10.1002/qua.560150606.

[ref125] Mestechkin M. Potential Energy Surface near the Hartree–Fock Instability Threshold. J. Mol. Struct.: THEOCHEM 1988, 181, 231 10.1016/0166-1280(88)80489-5.

[ref126] Trail J. R.; Towler M. D.; Needs R. J. Unrestricted Hartree–Fock theory of Wigner crystals. Phys. Rev. B 2003, 68, 045107 10.1103/PhysRevB.68.045107.

[ref127] Burton H. G. A. Hartree–Fock critical nuclear charge in two-electron atoms. J. Chem. Phys. 2021, 154, 111103 10.1063/5.0043105. 33752345

[ref128] Gilmore R. Catastrophe Theory for Scientists and Engineers, 1st ed.; Dover Publications: New York, 1993.

[ref129] Bauschlicher C. W. Jr.; Taylor P. R. Benchmark full configuration-interaction calculations on H_2_O, F, and F^–^. J. Chem. Phys. 1986, 85, 2779 10.1063/1.451034.

[ref130] Bauschlicher C. W. Jr.; Taylor P. R. A full CI treatment of the ^1^A_1_–^3^B_1_ separation in methylene. J. Chem. Phys. 1986, 85, 6510 10.1063/1.451431.

[ref131] Schaefer H. F. Methylene: A Paradigm for Computational Quantum Chemistry. Science 1986, 231, 1100 10.1126/science.231.4742.1100. 17818539

[ref132] Zarotiadis R. A.; Burton H. G. A.; Thom A. J. W. Towards a Holomorphic Density Functional Theory. J. Chem. Theory Comput. 2020, 16, 7400 10.1021/acs.jctc.0c00822. 33211476

[ref133] Huynh B. C.; Thom A. J. W. Symmetry in Multiple Self-Consistent-Field Solutions of Transition-Metal Complexes. J. Chem. Theory Comput. 2020, 16, 904 10.1021/acs.jctc.9b00900. 31820967

[ref134] Jiménez-Hoyos C. A.; Henderson T. M.; Scuseria G. E. Generalized Hartree–Fock Description of Molecular Dissociation. J. Chem. Theory Comput. 2011, 7, 2667 10.1021/ct200345a. 26605457

[ref135] Weser O.; Liebermann N.; Kats D.; Alavi A.; Li Manni G. Spin Purification in Full-CI Quantum Monte Carlo via a First-Order Penalty Approach. J. Phys. Chem. A 2022, 126, 2050 10.1021/acs.jpca.2c01338. 35298155 PMC8978180

[ref136] Paldus J. Matrix elements of unitary group generators in many-fermion correlation problem. I. tensorial approaches. J. Math. Chem. 2021, 59, 1 10.1007/s10910-020-01172-9.

[ref137] Paldus J. Matrix elements of unitary group generators in many-fermion correlation problem. II. Graphical methods of spin algebras. J. Math. Chem. 2021, 59, 37 10.1007/s10910-020-01173-8.

[ref138] Paldus J. Group theoretical approach to the configuration interaction and perturbation theory calculations for atomic and molecular systems. J. Chem. Phys. 1974, 61, 5321 10.1063/1.1681883.

[ref139] Shavitt I. Graph theoretical concepts for the unitary group approach to the many-electron correlation problem. Int. J. Quantum Chem. 1977, 12, 131 10.1002/qua.560120819.

[ref140] Brooks B. R.; Schaefer H. F. III. The graphical unitary group approach to the electron correlation problem. Methods and preliminary applications. J. Chem. Phys. 1979, 70, 5092 10.1063/1.437351.

[ref141] Fales B. S.; Martínez T. J. Fast transformations between configuration statefunction and Slater determinant basesfor direct configuration interaction. J. Chem. Phys. 2020, 152, 164111 10.1063/5.0005155. 32357800

[ref142] Dobrautz W.; Weser O.; Bogdanov N. A.; Alavi A.; Li Manni G. Spin-Pure Stochastic-CASSCF via GUGA-FCIQMC Applied to Iron–Sulfur Clusters. J. Chem. Theory Comput. 2021, 17, 5684 10.1021/acs.jctc.1c00589. 34469685 PMC8444347

[ref143] Thom A. J. W.; Head-Gordon M. Hartree–Fock solutions as a quasidiabatic basis for nonorthogonal configuration interaction. J. Chem. Phys. 2009, 131, 124113 10.1063/1.3236841. 19791858

[ref144] Bauschlicher C. W.; Langhoff S. R. Full configuration-interaction study of the ionic-neutral curve crossing in LiF. J. Chem. Phys. 1988, 89, 4246 10.1063/1.455702.

[ref145] Malrieu J.-P.; Heully J.-L.; Zaitsevskii A. Multiconfigurational second-order perturbative methods: Overview and comparison of basic properties. Theor. Chim. Acta 1995, 90, 167 10.1007/BF01113846.

[ref146] Thom R. Structural Stability and Morphogenesis, 1st ed.; Westview Press: Reading, MA, 1994.

