# Supplementary material for: Excited States, Symmetry Breaking, and Unphysical Solutions in State-Specific CASSCF Theory
Source: J Phys Chem A. 2023 May 4;127(20):4538–52. doi: 10.1021/acs.jpca.3c00603 (PMC10226133; doi:10.1021/acs.jpca.3c00603)
Supplement: Supplementary file 1 — jp3c00603_si_001.pdf [file jp3c00603_si_001.pdf]

Excited States, Symmetry Breaking, and Unphysical Solutions in State-Specific CASSCF Theory: Supporting Information

Antoine Marie[1,2] and Hugh G. A. Burton*[1,a)]

[1)] *Physical and Theoretical Chemical Laboratory, Department of Chemistry, University of Oxford, Oxford, OX1 3QZ, U.K.*

[2)] *Present address: Laboratoire de Chimie et Physique Quantiques (UMR 5626), Université de Toulouse, CNRS, UPS, 31062, Toulouse, France*

(Dated: 20 April 2023)

S1. Deriving the gradient and second-derivative of the CASSCF energy

S1.1. Notation and reduced density matrices

Here, we provide explicit expressions for the gradient and Hessian of the CASSCF energy, which are required for quasi-Newton optimisation algorithms. These expressions are derived in detail in Refs. 1 and 2, but we include this pedagogical discussion for completeness. In what follows, the indices m, n, p, q, r, s correspond to arbitrary spatial orbitals, i, j, k, l correspond to occupied orbitals, a, b, c, d correspond to virtual orbitals, and t, v, x, y correspond to active orbitals. Furthermore, we employ the ground-state one- and two-body reduced densities,

$$\gamma_{pq} = \sum_{\sigma} \langle \Psi_0 | \hat{a}^{\dagger}_{q\sigma} \hat{a}_{p\sigma} | \Psi_0 \rangle \,, \tag{S1a}$$

$$\Gamma_{pqrs} = \sum_{\sigma\tau} \langle \Psi_0 | \hat{a}^{\dagger}_{p\sigma} \hat{a}^{\dagger}_{r\tau} \hat{a}_{s\tau} \hat{a}_{q\sigma} | \Psi_0 \rangle \,, \tag{S1b}$$

and the transition density matrices

$$\gamma^{K}_{pq} = \sum_{\sigma} \langle \Psi_0 | \hat{a}^{\dagger}_{q\sigma} \hat{a}_{p\sigma} | \Psi_K \rangle \,, \tag{S2a}$$

$$\Gamma^{K}_{pqrs} = \sum_{\sigma\tau} \langle \Psi_0 | \hat{a}^{\dagger}_{p\sigma} \hat{a}^{\dagger}_{r\tau} \hat{a}_{s\tau} \hat{a}_{q\sigma} | \Psi_K \rangle \,. \tag{S2b}$$

Explicit expressions for the non-zero matrix elements can be simplified depending on the types of orbitals involved, giving expressions for the ground-state density matrices as

$$\gamma_{ij} = 2\delta_{ij} \tag{S3}$$

and

$$\Gamma_{ijkl} = 4\delta_{ij}\delta_{kl} - 2\delta_{il}\delta_{kj}, \qquad \Gamma_{ijtv} = 2\delta_{ij}\,\gamma_{vt}, \qquad \text{and} \qquad \Gamma_{ivtj} = -\delta_{ij}\,\gamma_{vt}. \tag{S4}$$

where the purely active components γ_{tv} and Γ_{tvxy} cannot be simplified beyond Eq. S1. The only non-zero component of the one-body transition density matrix is γ^{K}_{tv}. In addition to the Γ^{K}_{tvxy} components, the non-zero terms of the two-body transition density matrix include

$$\Gamma^{K}_{ijtv} = 2\delta_{ij}\,\gamma^{K}_{vt}, \qquad \text{and} \qquad \Gamma^{K}_{ivtj} = -\delta_{ij}\,\gamma^{K}_{vt}. \tag{S5}$$

S1.2. Gradient terms

The gradient can be divided into the orbital components g^{o} and the CI components g^{c} that are defined in the main text. The CI components are given by the Hamiltonian matrix elements in the configuration space, that is

$$g^{\mathrm{c}}_{K} = 2\,\langle \Psi_0 | \hat{H} | \Psi_K \rangle \,. \tag{S6}$$

The orbital components are given by

$$g^{\mathrm{o}}_{mn} = \langle \Psi_0 | [\hat{H}, \hat{E}^{-}_{mn}] | \Psi_0 \rangle \tag{S7}$$

[a)] Electronic mail: hgaburton@gmail.com

where

$$\langle\Psi_0|[\hat{H},\hat{E}_{mn}]|\Psi_0\rangle = \sum_p \left(h_{mp}\gamma_{pn} + \gamma_{np}h_{pm}\right) + \sum_{prs}\left[(pm|rs)\Gamma_{pnrs} + (mp|rs)\Gamma_{nprs}\right], \tag{S8}$$

and we have introduced the singlet excitation operator

$$\hat{E}_{mn} = \sum_{\sigma\in\{\uparrow,\downarrow\}} \hat{a}^\dagger_{m\sigma}\hat{a}_{n\sigma} \tag{S9}$$

with its anti-symmetrized variant $\hat{E}^-_{mn} = \hat{E}_{mn} - \hat{E}_{nm}$.

S1.3. Hessian terms

Similarly, we can divide the Hessian $\boldsymbol{Q}$ into three components: $\boldsymbol{Q}^{\mathrm{cc}}$, $\boldsymbol{Q}^{\mathrm{oo}}$ and $\boldsymbol{Q}^{\mathrm{oc}}$ (where $H^{\mathrm{co}} = (\boldsymbol{Q}^{\mathrm{oc}})^\dagger$). The CI-CI components are given by the Hamiltonian matrix elements within the active configuration space shifted by the current energy E_0, giving

$$Q^{\mathrm{cc}}_{K,L} = 2\,\langle\Psi_K|\hat{H} - E_0|\Psi_L\rangle\,. \tag{S10}$$

The off-diagonal components corresponding to the orbital-CI matrix elements are given by

$$Q^{\mathrm{oc}}_{mn,K} = \langle\Psi_0|[\hat{H},\hat{E}^-_{mn}]|\Psi_K\rangle \tag{S11}$$

where the constituent transition matrix elements are computed as

$$\langle\Psi_K|[\hat{H},\hat{E}_{mn}]|\Psi_0\rangle = \sum_p \left(h_{mp}\gamma^K_{pn} + \gamma^K_{np}h_{pm}\right) + \sum_{prs}\left[(pm|rs)\Gamma^K_{pnrs} + (mp|rs)\Gamma^K_{nprs}\right]. \tag{S12}$$

The final orbital-orbital term is given by

$$Q^{\mathrm{oo}}_{pq,rs} = \frac{1}{2}\Big(\langle\Psi_0|[[\hat{H},\hat{E}^-_{pq}],\hat{E}^-_{rs}]|\Psi_0\rangle + \langle\Psi_0|[[\hat{H},\hat{E}^-_{rs}],\hat{E}^-_{pq}]|\Psi_0\rangle\Big). \tag{S13}$$

While a full derivation using the results of Ref. 2 is lengthy, the non-redundant blocks are:

- Virtual-Core, Virtual-Core:

$$Q^{\mathrm{oo}}_{ai,bj} = 4[4(ai|bj) - (ab|ij) - (aj|bi)] + 4\delta_{ij}(F^{\mathrm{C}}_{ab} + F^{\mathrm{A}}_{ab}) - 4\delta_{ab}(F^{\mathrm{C}}_{ij} + F^{\mathrm{A}}_{ij}) \tag{S14}$$

- Virtual-Core, Virtual-Active:

$$Q^{\mathrm{oo}}_{ai,bt} = 2\sum_v \gamma_{tv}[4(ai|bv) - (av|bi) - (ab|vi)] - \delta_{ab}\left(\sum_v \gamma_{tv}F^{\mathrm{C}}_{iv} + 2(F^{\mathrm{C}}_{ti} + F^{\mathrm{A}}_{ti}) + \sum_{vxy}\Gamma_{tvxy}(vi|xy)\right) \tag{S15}$$

- Virtual-Core, Active-Core:

$$Q^{\mathrm{oo}}_{ai,tj} = 2\sum_v (2\delta_{tv} - \gamma_{tv})[4(ai|vj) - (av|ji) - (aj|vi)] - \delta_{ij}\left(\sum_v \gamma_{tv}F^{\mathrm{C}}_{av} - 4(F^{\mathrm{C}}_{at} + F^{\mathrm{A}}_{at}) + \sum_{vxy}\Gamma_{tvxy}(av|xy)\right) \tag{S16}$$

- Virtual-Active, Virtual-Active:

$$Q^{\mathrm{oo}}_{at,bu} = 2\sum_{vx}\Big(\Gamma_{tuvx}(ab|vx) + (\Gamma_{txvu} + \Gamma_{txuv})(ax|bv)\Big) - \delta_{ab}\left(\sum_v \Big(\gamma_{tv}F^{\mathrm{C}}_{uv} + \gamma_{uv}F^{\mathrm{C}}_{tv}\Big) + \sum_{vxy}\Big(\Gamma_{tvxy}(uv|xy) + \Gamma_{uvxy}(tv|xy)\Big)\right) + 2\gamma_{tu}F^{\mathrm{C}}_{ab} \tag{S17}$$

- Active-Core, Virtual-Active:

$$Q^{\mathrm{oo}}_{ti,au} = -2\sum_{vx}\Big(\Gamma_{tuvx}(ai|vx) + (\Gamma_{tvux} + \Gamma_{tvxu})(ax|vi)\Big) + 2\sum_v \gamma_{uv}\Big(4(av|ti) - (ai|tv) - (at|vi)\Big) - 2\gamma_{tu}F^{\mathrm{C}}_{ia} + \delta_{tu}(F^{\mathrm{C}}_{ai} + F^{\mathrm{A}}_{ai}) \tag{S18}$$

- Active-Core, Active-Core:

$$
\begin{aligned}
G_{ti,uj} &= 4\sum_{v}(\delta_{tv}-\gamma_{tv})\Big(4(vi|uj)-(ui|vj)-(uv|ij)\Big)+2\sum_{vx}\Big(\Gamma_{utvx}(vx|ij)+(\Gamma_{uxvt}+\Gamma_{uxtv})(vi|xj)\Big)\\
&\quad+2\gamma_{tu}F^{\mathrm{C}}_{ij}-2\delta_{ij}\left(\sum_{vxy}\Gamma_{tvxy}(uv|xy)+\sum_{v}\gamma_{uv}F^{\mathrm{C}}_{tv}\right)+4\delta_{ij}(F^{\mathrm{C}}_{tu}+F^{\mathrm{A}}_{tu})-4\delta_{tu}(F^{\mathrm{C}}_{ij}+F^{\mathrm{A}}_{ij}) \qquad \text{(S19)}\\
Q^{\mathrm{oo}}_{ti,uj} &= \frac{1}{2}\Big(G_{ti,uj}+G_{uj,ti}\Big).
\end{aligned}
$$

Here, we have introduced the inactive (core) and active Fock matrices[2]

$$
F^{\mathrm{C}}_{mn} = h_{mn}+\sum_{i}\left[2(mn|ii)-(mi|in)\right] \qquad \text{and} \qquad F^{\mathrm{A}}_{mn}=\sum_{tv}\gamma_{tv}\left[(mn|tv)-\frac{1}{2}(mv|tn)\right]. \tag{S20}
$$

S2. Eigenvector-following for saddle point optimisation

We employ the eigenvector-following technique to target stationary points with an arbitrary Hessian index. While Section 6.2.1 of Ref. 3, and references therein, describe this method in detail, here we summarise the salient points for completeness and provide the details of our particular implementation.

Eigenvector-following works in the eigenbasis for the Hessian matrix $\boldsymbol{Q}$ with eigenvalues ϵ_{μ}. In this basis, the components of the Newton–Raphson step $\boldsymbol{x} = -\boldsymbol{Q}^{-1}\boldsymbol{g}$, with gradient $\boldsymbol{g}$, and the change in energy ΔV are given by

$$
x_{\mathrm{NR},\mu} = -\frac{g_{\mu}}{\epsilon_{\mu}} \qquad \text{and} \qquad \Delta V_{\mathrm{NR}} = -\sum_{\mu}\frac{|g_{\mu}|^{2}}{2\epsilon_{\mu}}. \tag{S21}
$$

Contributions with $\epsilon_{\mu} > 0$ or $\epsilon_{\mu} < 0$ lower or raise the energy, respectively. To drive the optimisation towards a particular type of Hessian index, eigenvector-following artificially modifies the sign of these components. In particular, the components of the quasi-Newton eigenvector-following step are defined as

$$
x_{\mathrm{QN},\mu} = \frac{\pm 2g_{\mu}}{|\epsilon_{\mu}|\left(1+\sqrt{1+\left|2g_{\mu}/\epsilon_{\mu}\right|^{2}}\right)}, \tag{S22}
$$

where a positive or negative step gives an uphill or downhill step, respectively. This expression reduces to the Newton–Raphson step in the $g_{\mu} \to 0$ limit and can be understood by imposing constraints on each component using Lagrange multipliers.[3] When an index-n saddle point is targeted in this work, the components corresponding to the lowest n eigenvalues of the Hessian are chosen to be downhill directions.

Furthermore, we employ a dogleg trust radius technique to control the step length and improve the local convergence behaviour. A trust radius method works by defining a region with radius ρ around the current point in which a quadratic approximation to the objective function is considered to be accurate. The next step is chosen by optimising the objective function within this trust region, known as the sub-problem. In practice, approximate solutions to the sub-problem are required, and we employ the dogleg method (see Section 4.1 of Ref. 4). This method requires an analogue of the steepest descent direction for saddle point optimisation, which we define as

$$
x_{\mathrm{SD},\mu} = \pm g_{\mu}, \tag{S23}
$$

where the positive and negative sign for each component are chosen in the same way as the quasi-Newton step [Eq. (S22)]. Defining an unconstrained step which optimises the energy along the steepest descent direction as

$$
\boldsymbol{x}_{\mathrm{U}} = -\frac{\boldsymbol{g}^{\dagger}\boldsymbol{x}_{\mathrm{SD}}}{\boldsymbol{x}_{\mathrm{SD}}^{\dagger}\boldsymbol{Q}\boldsymbol{x}_{\mathrm{SD}}}\boldsymbol{x}_{\mathrm{SD}}, \tag{S24}
$$

and using the eigenvector-following quasi-Newton step $\boldsymbol{x}_{\mathrm{QN}}$, the optimal dogleg step $\boldsymbol{x}$ is then given by

$$
\boldsymbol{x} = \begin{cases} \boldsymbol{x}_{\mathrm{QN}} & \text{if } |\boldsymbol{x}_{\mathrm{QN}}| \le \rho \\ \frac{\rho}{|\boldsymbol{x}_{\mathrm{U}}|}\boldsymbol{x}_{\mathrm{U}} & \text{if } |\boldsymbol{x}_{\mathrm{U}}| \ge \rho \\ \boldsymbol{x}_{\mathrm{U}} + \tau\left(\boldsymbol{x}_{\mathrm{QN}} - \boldsymbol{x}_{\mathrm{U}}\right) & \text{otherwise} \end{cases} \tag{S25}
$$

where

$$\tau = \frac{-b + \sqrt{b^2 - 4ac}}{2a}, \qquad a = \left|\boldsymbol{x}_{\mathrm{QN}} - \boldsymbol{x}_{\mathrm{U}}\right|^2, \qquad b = 2\boldsymbol{x}_{\mathrm{U}} \cdot (\boldsymbol{x}_{\mathrm{QN}} - \boldsymbol{x}_{\mathrm{U}}), \qquad c = |\boldsymbol{x}_{\mathrm{U}}|^2 - \rho^2. \tag{S26}$$

The trust radius is then updated by comparing the ratio of the actual energy change ΔE to that predicted by the quadratic model $\Delta E_{\mathrm{model}}$. The trust radius is halved for $\Delta E/\Delta E_{\mathrm{model}} < 0.25$, and doubled if $\Delta E/\Delta E_{\mathrm{model}} > 0.75$ and $|\boldsymbol{x}_{\mathrm{DL}}| = \rho$. Otherwise, the trust radius is not deemed to be interfering with the optimisation and is left unchanged. Finally, a step is rejected if ΔE and $\Delta E_{\mathrm{model}}$ have different signs. We find that an initial trust radius of 0.15 provides adequate optimisation behaviour.

S3. Mode-controlled Newton–Raphson optimisation

Once a stationary point has been identified, it can be followed along a potential energy surface by using the old orbital and CI coefficients as a guess at the new geometry. Since the Hessian index can change at different geometries, we use a standard Newton–Raphson optimisation to perform this optimisation. The Newton–Raphson step is identified by solving a quadratic Taylor series expansion to the energy around a given point, giving

$$\boldsymbol{x}_{\mathrm{NR}} = -\boldsymbol{Q}^{-1}\boldsymbol{g} \tag{S27}$$

for the gradient $\boldsymbol{g}$ and Hessian matrix of second derivatives $\boldsymbol{Q}$. The dogleg method (described in Section S2) cannot be used as there is no way of defining a "steepest descent" step for saddle point optimisation without knowing the target Hessian index. Instead, we simply truncate the components of $\boldsymbol{x}_{\mathrm{NR}}$ depending on their magnitude along each eigen-direction of the $\boldsymbol{Q}$, giving

$$x_\mu = \min\left(|x_{\mathrm{NR},\mu}|\,, \rho\right) \frac{x_{\mathrm{NR},\mu}}{|x_{\mathrm{NR},\mu}|}. \tag{S28}$$

The trust radius ρ is then updated using the same strategy described in Section S2. Similar mode-controlled Newton–Raphson optimisation algorithms have been reported multiple times in the past.[1,5–7]

References

[1] Olsen, J.; Yeager, D. L.; Jørgensen, P. Optimization and Characterization of a Multiconfigurational Self-Consistent Field (MCSCF) State. In *Adv. Chem. Phys.*; John Wiley and Sons, Ltd, 1983; pp 1–176.

[2] Helgaker, T.; Jørgensen, P.; Olsen, J. *Molecular Electronic-Structure Theory*; John Wiley & Sons: Chichester, 2000.

[3] Wales, D. J. *Energy Landscapes: Applications to Clusters, Biomolecules and Glasses*; Cambridge University Press: Cambridge, 2004.

[4] Nocedal, J.; Wright, S. *Numerical Optimization*; Springer-Verlag: New York, 2006.

[5] Olsen, J.; Jørgensen, P.; Yeager, D. L. Multiconfigurational Hartree–Fock studies of avoided curve crossing using the Newton–Raphson technique. *J. Chem. Phys.* **1982**, *76*, 527.

[6] Golab, J. T.; Yeager, D. L.; Jørgensen, P. Proper characterization of MC SCF stationary points. *Chem. Phys.* **1983**, *78*, 175.

[7] Hoffmann, M. R.; Sherrill, C. D.; Leininger, M. L.; Schaefer III, H. F. Optimization of MCSCF excited states using directions of negative curvature. *Chem. Phys. Lett.* **2002**, *355*, 183.
